# Uncovering the NAFLD burden in people living with HIV from high‐ and middle‐income nations: a meta‐analysis with a data gap from Subsaharan Africa

**DOI:** 10.1002/jia2.26072

**Published:** 2023-03-16

**Authors:** Ramiro Manzano‐Nunez, Jesús Rivera‐Esteban, Jordi Navarro, Juan Bañares, Elena Sena, Jörn M. Schattenberg, Jeffrey V. Lazarus, Adria Curran, Juan M. Pericàs

**Affiliations:** ^1^ Liver Unit, Internal Medicine Department Vall d'Hebron University Hospital Barcelona Spain; ^2^ Vall d'Hebron Institute for Research Barcelona Spain; ^3^ Faculty of Medicine Universitat Autònoma de Barcelona Barcelona Spain; ^4^ HIV Unit, Infectious Disease Department Vall d'Hebron University Hospital Barcelona Spain; ^5^ Metabolic Liver Disease Research Program I. Department of Medicine University Medical Center of the Johannes Gutenberg‐University Mainz Germany; ^6^ Barcelona Institute for Global Health (ISGlobal) Hospital Clínic University of Barcelona Barcelona Spain; ^7^ Faculty of Medicine and Health Sciences University of Barcelona Barcelona Spain; ^8^ CUNY Graduate School of Public Health and Health Policy New York New York USA; ^9^ Centros de Investigación Biomédica en Red Enfermedades Hepáticas y Digestivas (CIBERehd) Madrid Spain

**Keywords:** NAFLD, MAFLD, liver fibrosis, HIV, people living with HIV, HIV epidemiology

## Abstract

**Introduction:**

Non‐alcoholic fatty liver disease (NAFLD) has become a significant concern among people living with HIV (PLHIV), albeit its burden remains unclear. The primary objective of this systematic review (SR) and meta‐analysis (MA) was to estimate the prevalence of NAFLD and significant fibrosis in PLHIV. The secondary objective was to determine the risk factors for NAFLD among PLHIV.

**Methods:**

We searched MEDLINE and Scopus from inception to 30 December 2022 for peer‐reviewed studies that included PLHIV and reported the prevalence of NAFLD. MA of proportions was used to estimate the pooled prevalence of NAFLD and significant fibrosis. MA of pre‐calculated effect estimates examined risk factors for NAFLD in PLHIV.

**Results:**

We included 24 articles published between 2009 and 2022, encompassing 6326 PLHIV. The pooled prevalence of NAFLD was 38% (95% CI: 31–45%) with high heterogeneity (*I* ^2^ = 96.3%). The pooled prevalence of significant fibrosis was 13% (95% CI: 8–18%) with high heterogeneity (*I* ^2^ = 92.09%). Subgroup analyses showed a NAFLD prevalence of 40% (95% CI: 24–57%) in the United States, 33% (95% CI: 31–36) in Asia, 42% (95% CI: 24–61%) in Europe and 33% (95% CI: 29–37) in South America. When stratifying by income level, NAFLD was 39% (95% CI: 31–48) prevalent in PLHIV from high‐income economies and 34% in both upper‐middle‐income (95% CI: 31–37%) and lower‐middle‐income economies (95% CI: 28–41%). Higher body mass index (BMI) (OR = 1.32, 95% CI: 1.13–1.55; *I* ^2^ = 89.9%), increasing triglycerides (OR = 1.48, 95% CI: 1.22–2.79; *I* ^2^ = 27.2%) and dyslipidaemia (OR = 1.89, 95% CI: 1.32–2.71; *I* ^2^ = 15.5%) were all associated with higher risk‐adjusted odds of NAFLD in PLHIV.

**Discussion:**

The burden of NAFLD and significant fibrosis in PLHIV is significant. Therefore, targeted efforts to screen and diagnose NAFLD in this population are needed. Health services for PLHIV could include ways to target NAFLD risk factors, screen for liver disease and implement interventions to treat those with significant fibrosis or more advanced stages of liver disease. Taking no action to address NAFLD in PLHIV should not be an option.

**Conclusions:**

This SR and MA found a 38% NAFLD and 13% significant fibrosis prevalence in PLHIV. Increasing triglyceride levels, higher BMI values and dyslipidaemia were associated with higher risk‐adjusted odds of NAFLD among PLHIV.

## 1 INTRODUCTION

Non‐alcoholic fatty liver disease (NAFLD) has become a major global public health issue affecting more than 30% of adults worldwide [1, 2]. It is the leading aetiology of chronic liver disease globally and will likely become the main cause of end‐stage liver disease in the near future [3, 4]. Additionally, NAFLD progresses to non‐alcoholic steatohepatitis (NASH) in about 20% of cases [5]. NASH is a major cause of progression to cirrhosis and hepatocellular carcinoma [6, 7, 8], with the latter being the second foremost driver of years of life lost among all cancers [9]. Furthermore, it has been determined that NAFLD patients have a high burden of comorbidities and often experience reduced quality of life [10]. The high overall burden of the disease is associated with increasing socio‐economic costs [11].

Despite the high burden of NAFLD in the general population, the prevalence and predictors of NAFLD in specific patient populations, such as people living with HIV (PLHIV), remain unclear. Yet, evidence suggests that chronic liver disease is the second leading cause of non‐HIV‐related mortality in PLHIV and that NAFLD disproportionately affects this population [12]. Recently, an expert panel review examined current knowledge gaps regarding the comorbidity burden in PLHIV and highlighted NAFLD/NASH as a research priority, including determining their prevalence and exploring predictors of NAFLD in this population [13]. This is central to ensuring the long‐term wellbeing of PLHIV through person‐centric health [14, 15, 16], as set out by the World Health Organization in the Global Sector Strategy on HIV for the period 2022–2030 [17], which proposes a continuum of care prioritizing prevention, diagnosis, treatment and chronic care as the pillars to deliver health services to PLHIV [14]. Of particular interest is the integration of HIV services with services for comorbidities that PLHIV may present.

However, to prioritize those pillars and improve the integration of HIV care with other health services, it is paramount to measure the HIV‐comorbidity burden to design and implement actions more efficiently. Measuring the burden of NAFLD in PLHIV will help us understand the potential threat metabolic‐associated fatty liver disease puts on this population. So far, however, efforts to quantify the NAFLD burden in PLHIV have been based on a few multicentre studies or data from particular settings. In 2017, Maurice et al. [18] synthesized the available data on NAFLD in PLHIV from the literature and estimated a pooled NAFLD prevalence of 35%. However, this prevalence estimation was based on data from five studies. Since then, the literature on the subject has expanded, which has increased the stakeholders’ awareness of NAFLD in priority populations such as PLHIV.

Synthesizing the growing body of literature can generate fresh insights and quantitative estimates of the burden of NAFLD in PLHIV. In this scenario, proportion meta‐analysis (MA) methods [19, 20] provide a means of getting a reliable and precise estimate of disease frequency. Therefore, serving as a convenient tool to appraise the burden of NAFLD in PLHIV. Unlike traditional MAs, used to examine the effects of interventions or study associations and thus aimed to calculate pooled estimates of effect size (i.e. risk ratio (RR), odds ratio [OR], risk difference and mean difference), an MA of proportions allows for pooling prevalence estimates under the assumption that prevalence follows a binomial distribution (number of events in a sample) [19, 20]. These methods have suffered significant improvements in recent years [20] and are now widely used to obtain disease frequency estimates using data from different settings across various disciplines [21, 22, 23].

The primary objective of this systematic review (SR) and MA was to estimate the prevalence of NAFLD and significant fibrosis in PLHIV. The secondary objective was to determine the risk factors for NAFLD among PLHIV.

## 2 METHODS

The present SR was conducted following the recommendations from the Cochrane Handbook of Systematic Reviews of interventions [24] and the Preferred Reporting Items for Systematic Reviews and Meta‐Analysis (PRISMA) guidelines [25].

Although not registered in PROSPERO, a protocol prepared before the review kickoff was used as the guide to plan and carry out the SR.

### 2.1 Inclusion and exclusion criteria

We included all peer‐reviewed studies that included persons being positive for HIV and reported the proportion (prevalence) of patients with NAFLD. In addition, studies examining risk factors for NAFLD in PLHIV were also considered eligible for inclusion.

In cases of overlapping populations (i.e. studies conducted in the same hospital or during overlapping periods), the publication with the largest sample size or more appropriate for the objective of this SR was selected for inclusion.

We excluded studies that included patients with either evidence of hepatitis B or C co‐infection or significant alcohol use. If studies did not specify these as exclusion criteria in the methodology section, we reviewed the results to know if patients with these characteristics were included. We also excluded case reports, reviews and comments/editorials/viewpoints.

### 2.2 Outcomes

The prevalence (proportion) of NAFLD in PLHIV was the primary outcome of interest.

NAFLD was defined as the presence of significant steatosis demonstrated by a right upper quadrant ultrasound, computer tomographic (CT) scan, magnetic resonance imaging (MRI) techniques, vibration‐controlled transient elastography (VCTE)‐based controlled attenuation parameter (CAP) measurements or liver biopsy. If the studies reported the assessment of liver steatosis by any of the methods mentioned above, then the study was considered to inform the primary outcome.

Secondary outcomes included the prevalence (proportion) of significant fibrosis in PLHIV and risk factors of NAFLD in the study population.

Data about significant fibrosis were collected as reported in the included studies, and the cut‐off values for it were defined in each study. Although current EASL guidelines [26] recommend a cut‐off value of 8 kPa to define significant fibrosis on VCTE, the synthesis reported in SRs and MAs depends on the data reported in primary sources. Therefore, we had to base our quantitative synthesis on the data and definitions of NAFLD from the studies included in this SR. If available, we defined significant liver fibrosis as a VCTE cut‐off value equal to or higher than 8 kPa as in the current EASL practice recommendation [26].

Reported measures relating to patient characteristics and comorbidities were extracted from the included articles to assess risk factors for NAFLD in PLHIV. The extracted risk factors of NAFLD had to be the result of a multivariate logistic regression analysis reporting adjusted ORs and corresponding 95% confidence intervals (CIs). We did not pre‐specify particular risk factors; instead, we extracted those available in the included studies.

### 2.3 Search methods

Following established recommendations [27, 28, 29], a computerized database search strategy of the available literature was performed. The literature search was performed in MEDLINE and Scopus from inception to 30th December 2022. The search strategy was developed with keywords and synonyms related to the population of interest (PLHIV) and the exposure/outcome of interest (NAFLD). We screened the references from the included studies and previous reviews on the same topic [18]. Complete electronic search strategies for each database are available in Supplementary File 1.

### 2.4 Study selection

The results from the search strategies were imported into Rayyan [30]. Then, two authors (RM and ES) independently reviewed the titles and abstracts identified in the database searches and made an initial selection based on inclusion and exclusion criteria. Papers that appeared relevant for inclusion were retrieved as full texts and subsequently reviewed by two investigators (RMN and ES) who independently applied inclusion and exclusion criteria to full texts for final eligibility. As previously mentioned, we selected the publication with the largest sample size or more appropriate for the SR objectives in cases where papers were at risk of reporting results from overlapping populations.

### 2.5 Data collection process

We created a data extraction form in which we collected the following information from the included studies: author, year of publication, study design, period of data collection, region where the study took place, hospital where the study was conducted, the total number of patients, number of patients with the outcomes of interest, patient demographic and clinical characteristics, comorbidity information and relevant lab‐values. In addition, we collected the data on the outcomes of interest and, whenever available, the results from the multivariable logistic regression models examining NAFLD risk factors in PLHIV.

We also collected the inclusion and exclusion criteria reported in each study and synthesized this information in Table S1 available in Supplementary File 1.

### 2.6 Risk of bias

Each study's quality and internal validity were assessed using the JBI's Critical Appraisal Tools (https://jbi.global/critical‐appraisal‐tools) [31, 32]. These tools assess the methodological quality of each study to determine the level to which research takes into account the potential for bias in its design and planning [31, 32, 33]. The procedures by which the JBI's tools assess the methodological quality and the risk of bias are described elsewhere [31, 32, 33, 34].

The critical appraisal and quality assessment results and a full explanation of how it was carried out are available in Supplementary File 1 (Figures S1–S3).

We did not assess for publication bias because available methods (funnel plot) were developed for comparative MA and are unreliable for MA of proportions [35].

### 2.7 Data synthesis and MA

The information and data used for this SR were extracted as reported in each study and summarized descriptively.

We undertook two different MAs: 1. To estimate the prevalence of NAFLD and significant fibrosis among PLHIV; we performed an MA of proportions with the “metaprop” command in Stata [20]. 2. To estimate the risk factors for NAFLD in PLHIV, we used reported effect estimates whenever available (adjusted ORs resulting from a multivariable regression analysis exploring the risk factors for NAFLD in each study) and combined these effect estimates in a random effect MA with the “metan” function in Stata [36].

#### 2.7.1 Prevalence of NAFLD among PLHIV: MA of proportions

The prevalence (proportion) of NAFLD and significant fibrosis were obtained by dividing the number of patients with the outcomes of interest (***n***) by the total number of patients (***N***) from each study. Then, these proportions were pooled in MAs of proportions using the “metaprop” command [20]. This command provides statistical methods for binomial data: ***n/N***, where ***n*** denotes the number of individuals with the characteristic/outcome of interest, and ***N*** refers to the total number of individuals. In our analysis, ***n*** corresponded to the number of patients with the outcome of interest (NAFLD, significant fibrosis) and ***N*** was the total number of PLHIV included in each study.

Study‐specific proportions with 95% CIs were estimated. To this end, we enabled the variance‐stabilizing transformation of the proportions suggested by Freeman and Tukey [37] and estimated study‐specific CIs by computing score confidence intervals [20, 38, 39]. Then, based on the transformed values and their variance, a random‐effects (Dersimonian and Laird) MA was used to compute the pooled estimates (pooled prevalence). For these pooled estimates, their respective confidence intervals were determined using the Wald method [20].

Subgroup analyses were performed for the pooled NAFLD prevalence on available study‐level characteristics. Hence, we performed subgroup analyses stratified by region and country income level, NAFLD diagnostic method and study design. We also performed a subgroup analysis of the NAFLD prevalence in the studies that reported data on significant fibrosis. Income level was defined according to the *<World Bank Country and Lending Groups>* definitions, which classify countries by income levels as high‐income, upper‐middle‐income, lower‐middle‐income and low‐income economies.

#### 2.7.2 MA of risk factors

MAs of risk factors of NAFLD were performed using the “metan” command in Stata [20]. We reviewed the studies that assessed and reported the risk factors (reported in adjusted ORs with 95% CIs) for NAFLD among PLHIV resulting from a multivariable regression analysis (Table 3). The adjusted ORs and its 95% CI (resulting from a multivariable regression analysis) were used as the effect statistics for the MAs. When at least three (≥3) studies captured the same risk factor and reported an adjusted OR for the same risk‐factor variable, we combined these effect estimates in a random‐effects (DerSimonian and Laird) MA to account for inevitable variations in settings, populations and adjusted covariates. We combined and meta‐analysed adjusted ORs for similar variables intending to assess the strength of association between the reported risk factor and NAFLD in PLHIV. The results were reported in forest plots of the estimated effects of the included studies with a 95% CI. The Supplementary File (3.2 Commands for the meta‐analysis of risk factors) provides a detailed explanation of pooling adjusted ORs.

In both MAs (proportion and precalculated effect estimates), heterogeneity was evaluated using the *I* ^2^ test. *I* ^2^ values corresponded to low (*I* ^2^<25%), medium (*I* ^2^ = 25–75%) and high (*I* ^2^>75%) heterogeneity.

All statistical analyses were performed in Stata statistical software version 14. The Stata commands employed to perform the MAs are available in the Supplementary File.

## 3 RESULTS

We identified 1785 articles from the electronic database searches (time‐period: inception–30 December 2022), of which 61 were considered eligible for inclusion in our SR. After applying all inclusion and exclusion criteria to the full texts, 22 peer‐reviewed articles were included. In addition, we reviewed a seminal prior SR and MA by Maurice et al. [18] and included two additional references that fulfilled the review's inclusion/exclusion criteria. Twenty‐four articles (*n* = 24) were finally included [40, 41, 42, 43, 44, 45, 46, 47, 48, 49, 50, 51, 52, 53, 54, 55, 56, 57, 58, 59, 60, 61, 62, 63]. Table S2 in the Supplementary File lists a number of key articles that were excluded from the present SR (reasons for exclusion are provided in the Table).

**Figure 1:**
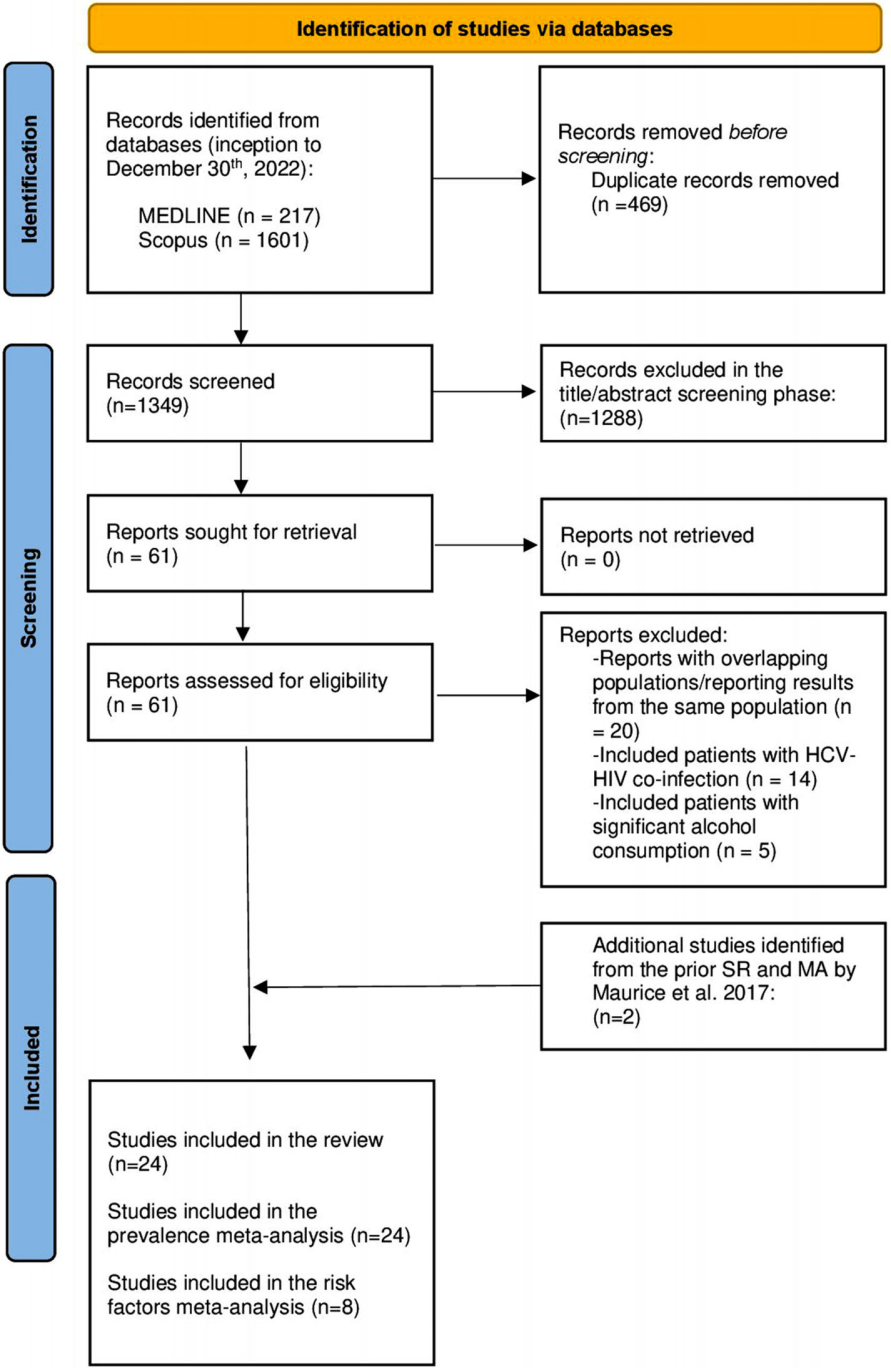
PRISMA diagram.

Of the 24 articles, eight (*n* = 8) reported a multivariate regression analysis of the factors associated with NAFLD in PLHIV and were included in the MA of risk factors. Figure 1 shows the PRISMA diagram for the selection of the studies.

### 3.1 Characteristics of included studies

The studies included in this SR were published between 2009 and 2022. As shown in Table 1, of the 24 included studies, six recruited participants in Asia and eight in Europe. Seven recruited patients in the United States. The two Latin‐American studies were from South America (Brazil). One was a multicentre study recruiting patients from Europe and North America (Sebastiani 2022) [56].

**Table 1:** Characteristics of included studies in the systematic review and meta‐analysis

Author/year	Country	City	Did the data originate from a well‐known‐relevant cohort?	Study design/analysis	Recruitment period	NAFLD evaluation method	NAFLD definition	PLHIV (*N*)	NAFLD (*N*)	SF definition	SF (*N*)
**Crum‐Cianflone 2009**	USA	San Diego, CA	No	Cross‐sectional	2006–2007	LUS	“Steatosis described as diffusion in hepatic echogenicity”	216	67	NR	NR
**Sterling 2013**	USA	Richmond, VA	No	Cross‐sectional	2007–2011	Biopsy	Biopsy >5%	14	9	NR	NR
**Nishijima 2014**	Japan	Tokyo	No	Cross‐sectional	2004–2013	LUS	NR	435	135	NR	NR
**G Lui 2016**	China	Hong Kong	No	Case–control	NR	MRI	H‐MRS ≥5%	80	23	LS≥7.0 kPa	11
**Kardashian 2017**	USA	Multicenter‐1	Yes: WIHS (NCT00000797)	Case–control	1994–2015	MRI	H‐MRS ≥5%	122	35	NR	NR
**Lombardi 2017**	UK	London	No	Cross‐sectional	2014	LUS	NR	156	47	NR	NR
**Price 2017**	USA	Multicenter‐2	Yes: MACS (NCT00046280)	Cross‐sectional	2010–2013	CT	Liver/spleen attenution ratio<1.0 on non‐contrast CT	329	44	NR	NR
**Aepfelbacher 2019**	USA	Bethesda, MA	Yes: Clinical Outcomes of People Who Acquired HIV in Early Life (NCT01656564)	Case–control	2016–2018	VCTE	CAP> = 248 dB/m	46	15	LS≥7.1 kPa	3
**Lallukka‐Brück 2019**	Finland	Helsinki	No	Retrospective cohort	2001–2019 (16 years follow‐up)	MRI	H‐MRS> = 5%	41	14	NR	NR
**Vujanović 2019**	Serbia	Novi Sad	No	Cross‐sectional	2016–2018	LUS	Increased liver echogenicity when compared to the parenchyma of the right kidney	88	37	NR	NR
**Ajmera 2020**	USA	San Diego, CA	No	Cross‐sectional	2016–2018	MRI	MRI‐PDFF ≥5%	70	56	NR	NR
**Ferri Pezzini 2020**	Brazil	Porto Alegre	No	Cross‐sectional	2016–2017	VCTE	CAP ≥238 dB/m	98	31	LS≥7.1 kPa	7
**Kaplan 2020**	USA	Boston, MA	No	Cross‐sectional	2010–2017	Biopsy or CT‐scan or ultrasound	Biopsy >5% OR validated radiographic criteria for CT/LUS	232	97	NR	NR
**Kirkegaard 2020**	Denmark	Copenhagen	Yes: COCOMO (NCT02382822)	Case–control	2015–2016	CT	CT‐liver attenuation <48 HUs	453	39	NR	NR
**Rasoulinejad 2020**	Iran	Tehran	No	Cross‐sectional	2018–2019	VCTE	NR	100	35	NR	NR
**Bischoff 2021**	Germany	Bonn	No	Cohort study	2013–2018 (24 months follow‐up)	VCTE	CAP ≥238 dB/m	319	109	NR	NR
**Fonseca de Almeida 2021**	Brazil	Rio de Janeiro	Yes: PROSPEC‐HIV (NCT02542020)	Cross‐sectional	2015–2019	VCTE	CAP ≥248 dB/m	451	152	LS≥7.1 kPa	72
**Jongraksak 2021**	Thailand	Bangkok	No	Cross‐sectional	2017–2018	VCTE	CAP ≥248 dB/m	150	48	LS≥7.1 kPa	9
**Liu Danping 2021**	China	Shangai	No	Cross‐sectional	2019–2020	VCTE	CAP ≥248 dB/m	361	136	LS≥10 kPa	30
**Arka De 2022**	India	Chandigarh	No	Cross‐sectional	NR	VCTE	CAP ≥251 dB/m	100	34	LS≥7.0 kPa	47
**Busca 2022**	Spain	Madrid	No	Cross‐sectional	2017–2018	Biopsy	Biopsy >5%	69	62	NR	NR
**Lemoine 2022**	Belgium, France and Germany	Paris, Bruxelles, Hambourg, Berlin, Düsseldorf and Hannover	Yes: ECHAM (NCT02093754)	Cross‐sectional	2014–2015	MRI	MRI‐PDFF> = 5%	402	257	NR	NR
**Michel 2022**	Germany	Mainz	Yes: FLASH (NCT04066608)	Cross‐sectional	2018–2021	VCTE	CAP ≥275 dB/m	245	85	LS≥8.2 kPa	16
**Sebastiani 2022**	Italy and Canada	Modena, Italy/Montreal, Canada/Palermo and Italy	Yes: LIVEHIV (CTN326 Canadian), MHMC (NR) and LHIVPA (NR)	Cross‐sectional	NR	VCTE	CAP ≥248 dB/m	1749	684	LS≥7.1 kPa	264

Abbreviations: CAP, controlled attenuation parameter; LS, liver stiffness; LUS, liver ultrasound; MRI, magnetic resonance imaging; NAFLD, non‐alcoholic fatty liver disease; NR, not reported; PLHIV, people living with HIV; SF, significant fibrosis; VCTE, vibration‐controlled transient elastography.

More than half of the papers included (*n* = 18, 75%) presented a cross‐sectional study design (Table 1). Four and two studies were case–control and cohort studies, respectively. Eight of the studies used data from cohorts registered in clinicaltrials.gov (Table 1).

The studies were comparable in terms of the populations included. All of them reported very similar inclusion and exclusion criteria, including PLHIV and excluding patients with known hepatitis B or C infection and significant/harmful alcohol use. Table S1, included in the Supplementary File, shows each study's objectives, inclusion, exclusion criteria, and relevant methodological characteristics.

### 3.2 Characteristics of participants

An overview of patients’ characteristics is shown in Table 2. We extracted and summarized data presented in each study from PLHIV.

**Table 2:** Characteristics of participants (PLHIV’ data from the studies included in the systematic review and meta‐analysis)

Author/year	PLHIV (*N*)	Age $	Females (*N*) %	CD4 count $	BMI $	Tcho $	TG $	AST $	ALT $	T2DM $	AHT %
**Crum‐Cianflone 2009**	216	39.6 (11.1)	12 (5.6)	535.2 (247.5)	26 (4.1)	185.9 (41.9)	172.1 (158.3)	NR	NR	11 (5.1)	49 (22.7)
**Sterling 2013**	14	45 (10)	4 (29)	614 (357)	29.9 (7.4)	172 (38)	235 (180)	76 (46)	94 (58)	3 (21)	NR
**Nishijima 2014**	435	40 (35–50)	29 (7)	349 (203–512)	22.1 (20.2–24.9)	175 (150–205)	162 (104–233)	25 (19–37)	26 (17–47)	22 (5)	86 (20)
**G Lui 2016**	80	54 (11)	6 (8)	503 (248)	23.6 (3.9)	185.6 (38.6)	159.29 (123.8–274.3)	26 (22–32)	86 (69–107)	39 (48.8)	33 (41.3)
**Kardashian 2017**	122	NR	58 (47.5)	NR	NR	NR	NR	NR	NR	NR	NR
**Lombardi 2017**	156	47.5 (8.5)	13 (8)	683 (4–1900)	NR	NR	NR	41 (22–299)	56 (29–372)	17 (11)	28 (18.2)
**Price 2017**	329	52 (47–57)	0 (0)	598 (438–776)	26 (23–29)	NR	130 (91–205)	24 (20–31)	25 (18–35)	39 (12)	152 (48)
**Aepfelbacher 2019**	46	27 (3.1)	28 (61)	605 (400)	27 (7)	165 (41)	109 (73)	25 (12)	23 (16)	NR	NR
**Lallukka‐Brück 2019**	41	41.9 (1.3)	7 (17)	NR	23.1 (0.5)	NR	168.1 (141.59–292.04)	32 (28–44)	30 (23–50)	0 (0)	NR
**Vujanović 2019**	88	39.94 (9.91)	0 (0)	NR	24.76 (3.58)	NR	290.27 (364.6)	25.1 (12.3)	30.5 (22.8)	NR	NR
**Ajmera 2020**	70	48.6 (10.2)	7 (10)	791 (688)	26.9 (4.6)	193.5	116.5 (48)	24.5 (20)	30.5 (19)	11 (15.7)	NR
**Ferri Pezzini 2020**	98	49 (11)	45 (46)	657.5 (118–208)	25.45 (23.6–28.2)	188.6 (34)	156 (118–218)	21 (17–26)	22 (16–30)	35 (35.7)	27 (27.6)
**Kaplan 2020**	232	54 (9)	55 (23.7)	NR	NR	NR	NR	NR	NR	44 (18.9)	98 (42.2)
**Kirkegaard 2020**	453	52.4 (46.8–61)	65 (14.3)	690 (520–884)	24.7 (22.4–27.5)	189.2 (162.1–220)	159.3 (115–247.8)	NR	26 (20–34)	33 (7.3)	NR
**Rasoulinejad 2020**	100	39.9 (9.5)	51 (51)	610	NR	NR	NR	NR	NR	1 (1)	NR
**Bischoff 2021**	319	47.5 (11.5)	72 (22.5)	NR	24.9 (4.5)	197.6 (43.1)	187.6 (148.3)	24.5 (14.2)	35.4 (18.1)	19 (6)	65 (20.4)
**Fonseca de Almeida 2021**	451	45 (36–53)	272 (60.3)	665 (421–881)	25 (23–29)	185 (158–219)	124 (84–171)	25 (20–33)	29 (23–43)	46 (10.2)	100 (22.2)
**Jongraksak 2021**	150	45.83 (9.54)	67 (44.7)	562 (217.7)	22.77 (3.57)	202.23 (42.78)	149.23 (98.92)	31.36 (10.27)	37.97 (34.14)	10 (6.7)	NR
**Liu danping 2021**	361	38 (31–48)	17 (4.7)	459 (327–633.5)	22.64 (21.04–24.73)	180.57 (155.82–205.7)	143.36 (95.58–238.05)	NR	29 (20–51)	29 (8.03)	36 (9.9)
**Arka De 2022**	100	36.8 (10.4)	35 (35)	NR	22.9 (4.3)	NR	187.2 (60.7)	NR	NR	4 (4)	8 (8)
**Busca 2022**	69	50 (44–54)	13 (9)	740 (593–930)	27 (24–30)	182 (159–203)	147 (97–213)	36 (28–43)	50 (41–77)	63 (44)	57 (39)
**Lemoine 2022**	402	55 (50–61)	62 (15)	603 (510–832)	27 (23.6–28.7)	NR	141.6 (97.3–221.2)	29 (23–37)	34 (24–50)	NR	NR
**Michel 2022**	245	52 (42–58)	71 (29)	727 (516–901)	25 (22.4–28.5)	202 (177–229)	133 (90–191)	26 (23–31)	23 (18–32)	27 (11)	75 (30.6)
**Sebastiani 2022**	1749	50.2 (10.4)	446 (25.5)	NR	NR	NR	NR	NR	NR	595 (34)	NR

Abbreviations: AHT, arterial hypertension; Tcho, total cholesterol; TG, triglycerides; T2DM, type 2 diabetes mellitus.

– $: Data presented in means/medians (SD/IQR); %: data presented in frequencies and percentages.

The studies included in this SR recruited 6326 PLHIV with a PLHIV sample size ranging from 14 to 1749 (median, 153; interquartile range, 84–345). Of these, 1435 (22.6%%) were females, and, as shown in Table 2, they tended to be young as all the reported means and medians of age were below 55 years. The reported values of the cluster of differentiation‐4 (CD4) lymphocyte count, BMI, lipid panel, hepatic enzymes, and the proportion of patients with diabetes and arterial hypertension are reported in Table 2.

### 3.3 NAFLD assessment

The methods to assess and the definitions with cut‐off values to diagnose NAFLD are detailed in Table 1.

VCTE was used in 10 (*n* = 10) studies. Hepatic steatosis was diagnosed with CAP values ≥248 dB/m in five of the studies and ≥238 dB/m in two. Michel (2022) and Arka De (2022) reported that hepatic steatosis was diagnosed using CAP values of 275 dB/m or higher and 251 dB/m or higher, respectively.

Five studies (*n* = 5) reported the use of MRI techniques (Table 1). In two of these studies, a liver fat content ≥5% in the MRI‐derived proton density fat fraction (MRI‐PDFF≥5%) was diagnostic of steatosis. In the other three (*n* = 3), steatosis was diagnosed by a liver fat content ≥5% in proton magnetic resonance spectroscopy (H‐MRS≥5%).

Four studies diagnosed steatosis through liver ultrasound. Two studies used biopsy, two CT scan and another (a multicentre study) reported the use of either biopsy, CT scan, or ultrasound to define steatosis (Table 1).

Nine of the 24 studies (including 3280 patients) reported data on significant fibrosis. In these studies (*n* = 9), significant fibrosis was diagnosed using VCTE to estimate liver stiffness (cut‐off values defining significant fibrosis are provided in Table 1). Among PLHIV, significant fibrosis was diagnosed in 459 (13.9%) of the 3280 participants from the nine studies reporting significant fibrosis data.

### 3.4 Risk of bias

Overall, the methodological quality of the included studies was deemed appropriate, and the research workflow appeared consistent throughout each report, meaning that the research aims were relevant and adequately defined, and the methodology used was pertinent to the objectives proposed. We did not find major red flags or significant methodological flaws in the studies included in the SR (Figures S1–S3).

### 3.5 Quantitative synthesis (MA)

#### 3.5.1 MA of NAFLD and significant fibrosis prevalence

**Figure 2:**
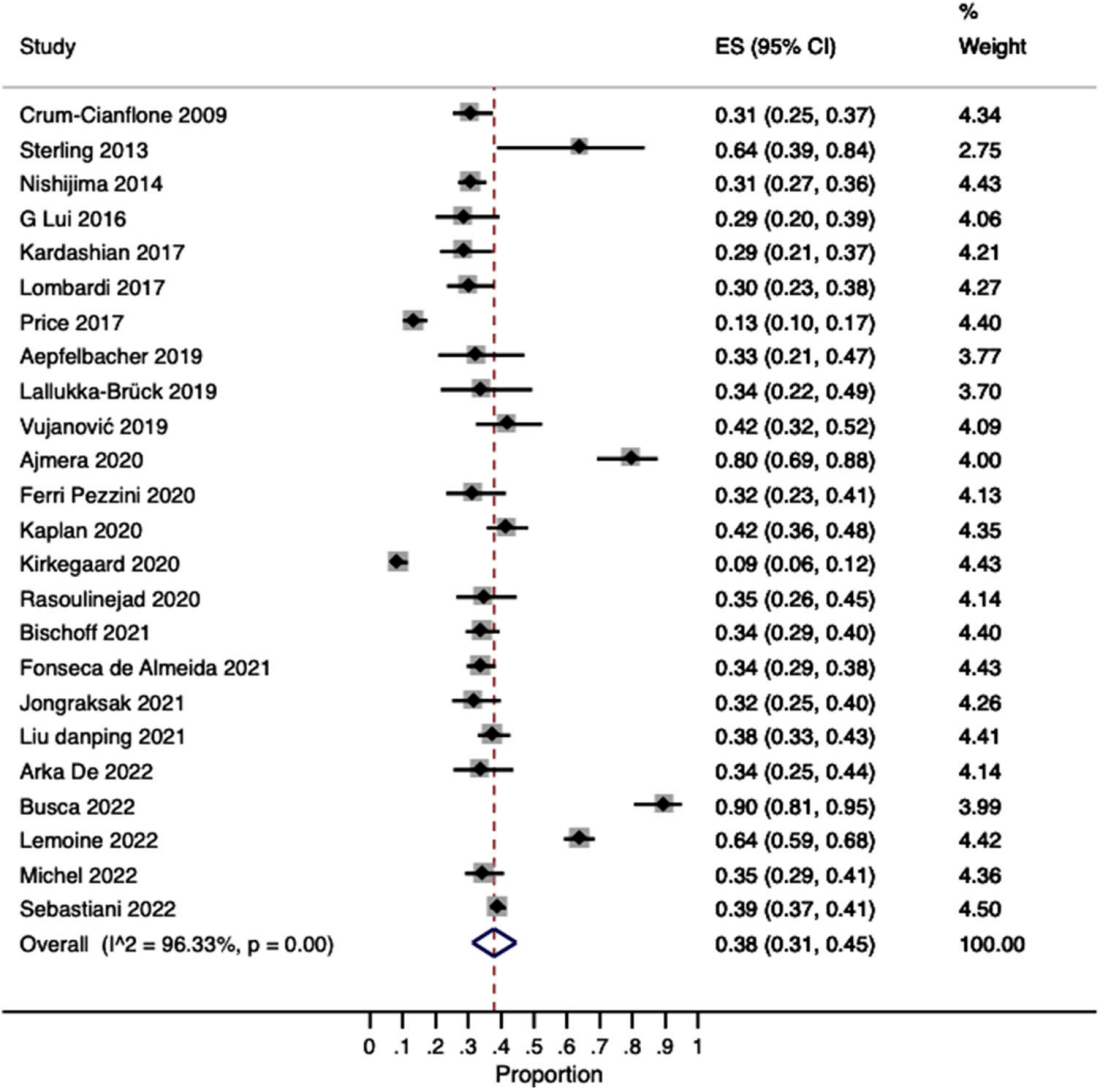
Pooled estimates of NAFLD prevalence.

**Figure 3:**
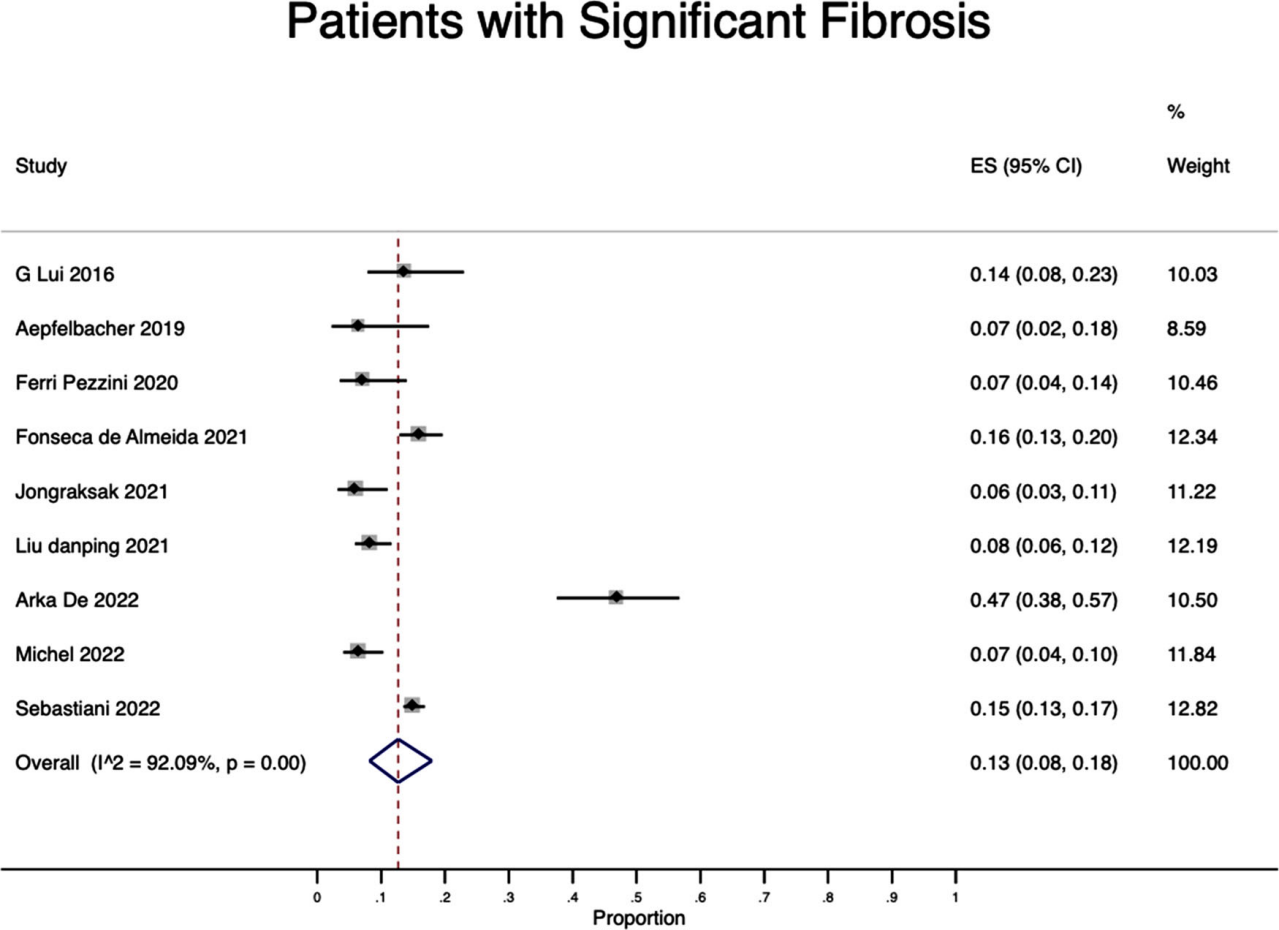
Pooled estimates of significant fibrosis prevalence.

We found that the pooled prevalence of NAFLD was 38% (95% CI: 31–45%) with high heterogeneity (*I* ^2^ = 96.3%) (Figure 2). Regarding significant fibrosis, we found a pooled prevalence of 13% (95% CI: 8–18%) with high heterogeneity (*I* ^2^ = 92.09%) (Figure 3).

#### 3.5.2 Subgroup analyses (MA of prevalence)

##### 3.5.2.1 Subgroup analyses by region and country income level

**Figure 4:**
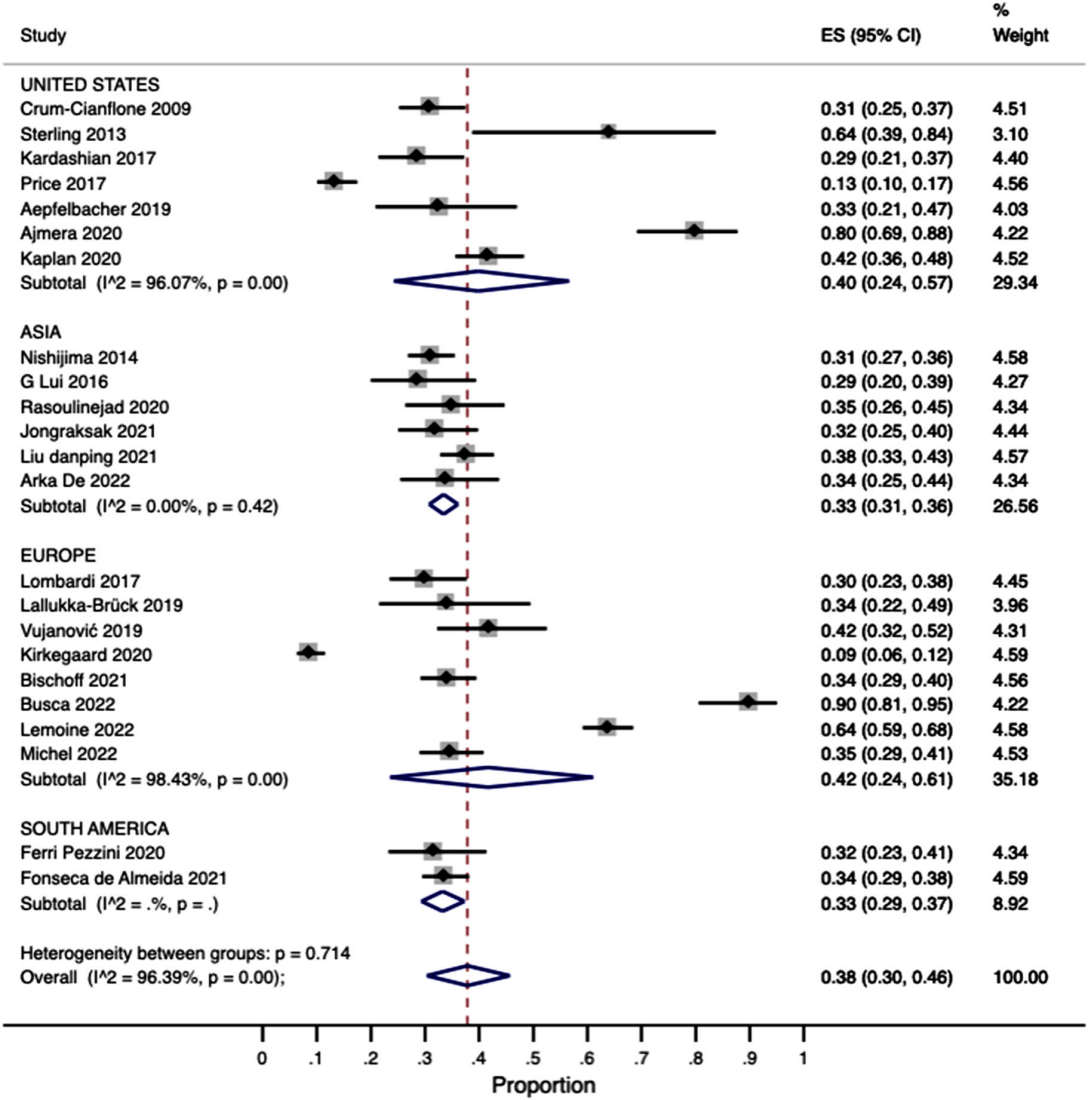
Subgroup analysis by region.

The subgroup analysis by region (Figure 4) showed that the pooled prevalence in Asia (data from six studies) and Europe (data from eight studies) was 33% (95% CI: 31–36%; *I* ^2^ = 0.0%) and 42% (95% CI: 24–61%; *I* ^2^ = 98.4%), respectively. The pooled prevalence in the United States (data from seven studies) was 40% (95% CI: 24–57; *I* ^2^ = 96.07%). Two studies reported data from South America with a pooled prevalence of 44% (95% CI: 29–59%; *I* ^2^ = not estimable).

**Figure 5:**
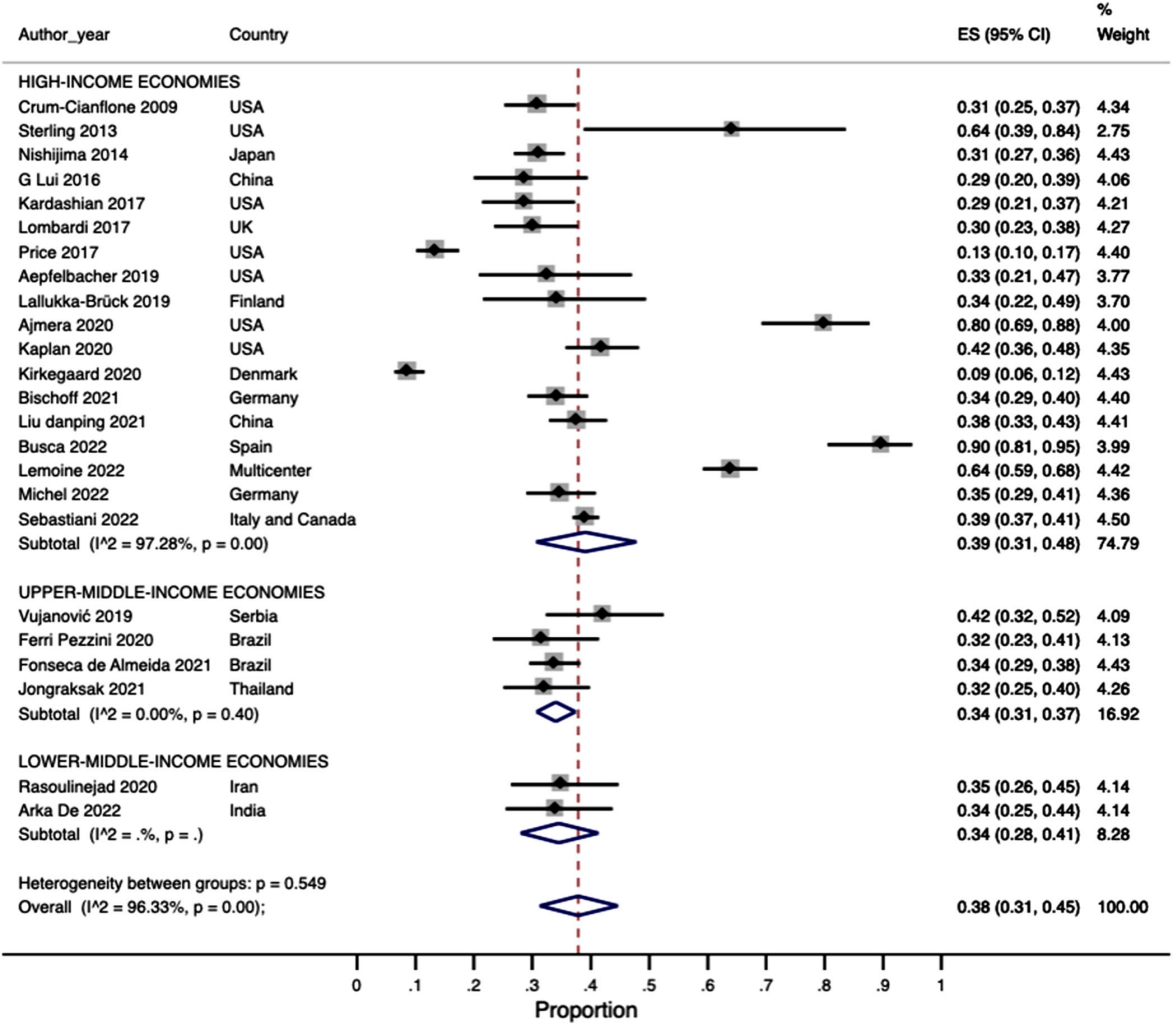
Subgroup analysis by country‐income level.

The subgroup analysis according to the World Bank's country income groups is shown in Figure 5. The pooled prevalence of NAFLD in high‐income countries was estimated in 39% (95% CI: 31–48%) with high heterogeneity (*I* ^2^ = 97.2%). In upper‐middle‐income and lower‐middle‐income countries, NAFLD was detected in 34% of PLHIV (Figure 5).

##### 3.5.2.2 Subgroup analyses by NAFLD diagnostic method

**Figure 6:**
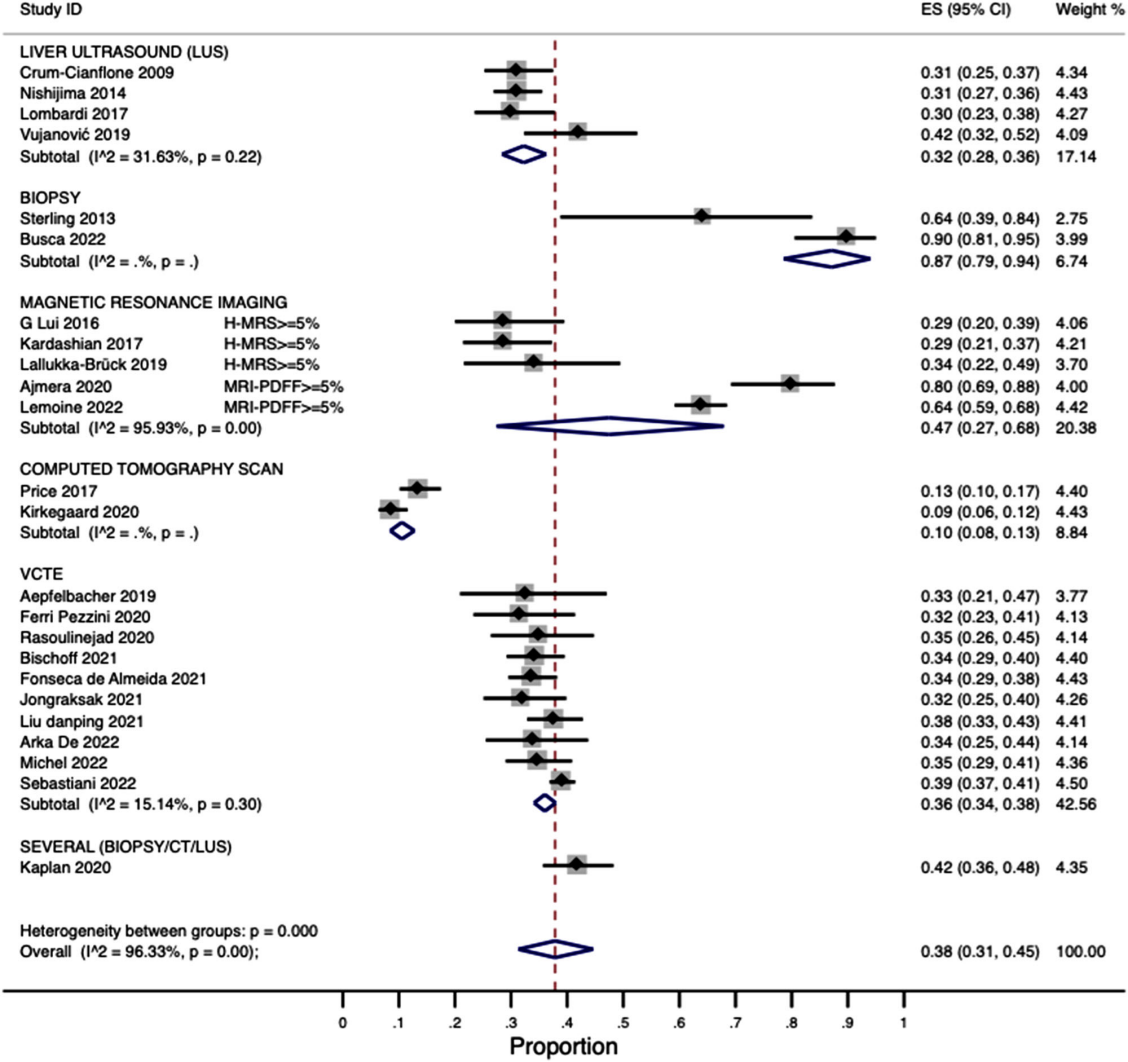
Subgroup analysis by diagnostic method.

We stratified the data by the NAFLD diagnostic method. The results from this analysis are shown in Figure 6. Ten studies reported the use of VCTE to detect/diagnose NAFLD. The pooled prevalence from these studies was 36% (95% CI: 34–38) with low heterogeneity (*I* ^2^ = 15.1%). The pooled prevalence from the studies (*n* = 4) using liver ultrasound was 32% (95% CI: 28–36) with medium heterogeneity (*I* ^2^ = 31.6%) (Figure 6). The pooled NAFLD prevalence from studies using MRI techniques (*n* = 5), CT‐scan (*n* = 2) and biopsy (*n* = 2) was 47% (*I* ^2^ = 95.9%), 10% (*I* ^2^ = not estimable) and 87% (*I* ^2^ = not estimable), respectively (Figure 6).

Five studies reported a CAP threshold for detecting NAFLD of ≥248 dB/m. The pooled prevalence from these studies was 36% (95% CI: 33–39%) with medium heterogeneity (*I* ^2^ = 43.3%). The pooled prevalence from the two studies with a CAP threshold for NAFLD diagnosis of ≥238 dB/m was 34% (95% CI: 29–38%) (heterogeneity not estimable). One study used a CAP threshold of ≥251 dB/m, finding a NAFLD prevalence of 34% (95% CI: 25–44). Another one that defined NAFLD as a CAP≥275 dB/m found a 34% (95% CI: 25–44) NAFLD prevalence (heterogeneity not estimable).

##### 3.5.2.3 Other subgroup analyses

We performed a subgroup analysis by the type of study design (Figure S4 available in the Supplementary File). The pooled prevalence from cross‐sectional studies (*n* = 18 studies) was 41% (95% CI: 35–49) with high heterogeneity (*I* ^2^ = 95.5%). In case–control (*n* = 4) and cohort studies (*n* = 2), NAFLD was detected in 23% (95% CI: 10–40%; *I* ^2^ = 93.9%) and 34% (95% CI: 29–39%; *I* ^2^ = not estimable) of PLHIV, respectively.

A second subgroup analysis of the studies that reported patients with significant fibrosis was performed. This analysis included nine studies and found a pooled NAFLD prevalence of 35% (95% CI: 33–38%) with medium heterogeneity (*I* ^2^ = 32.8%).

#### 3.5.3 MA of risk factors

We found eight (*n* = 8) studies reporting a multivariable regression analysis of the factors associated with NAFLD. These studies used logistic regression analysis, with NAFLD as the outcome variable and several independent variables (factors associated/predictors), to determine the risk factors for NAFLD among PLHIV. The ORs resulting from the analyses reported in each study are reported in Table 3.

**Table 3:** Logistic regression models from each study reporting the factors associated with NAFLD

	Variable	Reported aOR (95% CI)
**Crum‐Cianflone 2009**	Waist circumference, cm (per 10‐cm increment)	2.1 (1.6–2.8)
	Triglycerides, mg/dl (per 100 mg/dl increment)	1.2 (1.0–1.5)
	Race: African American	0.4 (0.2–1.1)
	Race: Hispanics	1.4 (0.6–3.3)
	Race: others (Filipino, Pacific Islander, other Asian or mixed)	1.7 (0.4–6.8)
	HDL, mg/dl	0.7 (0.5–1.0)
	Dyslipidaemia (on lipid‐lowering drugs)	1.3 (0.7–2.7)
	Years of HIV	1.1 (0.8–1.6)
	Staduvine history	0.9 (0.3–2.2)
**Nishijima 2014**	Male sex	1.95 (0.64–5.96)
	Age (per 1‐year difference)	1.005 (0.98–1.02)
	BMI, kg/m^2^ (per 1 kg/m^2^ increment)	1.19 (1.11–1.29)
	Dyslipidaemia (on lipid‐lowering drugs)	2.04 (1.18–3.53)
	ALT/AST ratio (per 1‐unit increment)	3.55 (2.12–5.94)
	Hypertension	0.95 (0.51–1.80)
	CD4 count (per cell)	1.001 (0.999–1.0002)
**Lui 2016** ^a^	Triglycerides, mmol/L	1.79 (1.12–2.86)
**Jongraksak 2020**	Age, years (per 1‐year difference)	1.076 (1.017–1.907)
	BMI, kg/m^2^ (per 1 kg/m^2^ increment)	1.596 (1.336–1.907)
	Dyslipidaemia (triglycerides>150 mg/dl)	3.72 (1.508–9.187)
**Kaplan 2020**	BMI, kg/m^2^ (per 1 kg/m^2^ increment)	1.10 (1.04–1.17)
	Hypertension	1.36 (0.71–2.60)
	Obstructive sleep apnea	1.89 (0.59–6.02)
	Smoking	0.74 (0.30–1.78)
	Dyslipidaemia (LDL>160 mg/dl)	1.67 (0.89–3.14)
	Diabetes	1.13 (0.51–2.52)
	CD4 count <200	4.67 (1.82–12.02)
	Diagnosis of HIV in the last 10 years	1.00 (0.96–1.03)
	Cardiovascular disease	3.08 (1.37–6.94)
**Kirkegaard 2020**	Age (per decade)	1.09 (0.64–1.86)
	Sex (female)	0.08 (0.01–0.78)
	Non‐Caucasian	1.08 (0.36–3.23)
	BMI, kg/m^2^ (per 1 kg/m^2^ difference/increment)	1.58 (1.35–1.85)
	Cholesterol (per 1 mM)	0.96 (0.58–1.57)
	Triglycerides (per 1 mM)	1.07 (0.80–1.44)
	Diabetes	3.43 (0.58–20.16)
	Plasma glucose (per 1 mM)	0.82 (0.58–1.16)
	ALT (per unit increment)	1.76 (1.31–2.37)
**Liu Danping 2021**	Age (per 1‐year difference)	1.01 (0.983–1.037)
	ALT, U/L (per unit increment)	1.015 (1.002–1.028)
	GGT, U/L (per unit increment)	1.000 (0.991–1.009)
	Waist/hip ratio (per 0.01)	0.944 (0.869–1.027)
	Waist/height ratio (per 0.01)	1.359 (1.219–1.515)
	Uric acid, μmol/L (per unit increment)	1.005 (1.002–1.009)
	CD4 count, cells/μl (per cell increment)	1.000 (0.999–1.001)
	Total cholesterol, mmol/L (per unit increment)	1.44 (1.05–1.97)
	LSM, kPa (per unit increment)	1.082 (0.9.0–1.259)
**Lemoine 2022**	ALT, U/L (per 5 units increment)	1.23 (1.16–1.31)
	CD4 cell count (per log2 unit)	4.04 (1.92–8.51)
	Ferritin, mmol/L (per unit increment)	1.05 (1.03–1.07)
	Triglycerides, mmol/L (per unit increment)	1.48 (1.18–1.84)
	Leptin ≥3.2 μg/L	2.12 (1.14–3.93)
	PNPLA3 rs738409 not C/C	1.84 (1.22–2.79)

Abbreviation: aOR, adjusted odds ratio.

^a^ The study by G. Lui 2016 reported the OR only for triglycerides; however, it was an adjusted odds ratio.

**Figure 7:**
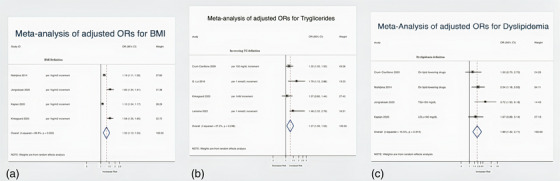
Meta‐analysis of risk factors. (a) Forest plot for BMI. (b) Forest plot for triglycerides. (c) Forest plot for dyslipidemia.

Figure 7 contains the MAs of adjusted ORs showing that higher BMI (four studies: OR = 1.32, 95% CI: 1.13–1.55; *I* ^2^ = 89.9%), increasing triglycerides (four studies: OR = 1.48, 95% CI: 1.22–2.79; *I* ^2^ = 27.2%) and dyslipidaemia (four studies: OR = 1.89, 95% CI: 1.32–2.71; *I* ^2^ = 15.5%) were all associated with significantly higher risk‐adjusted odds of NAFLD in PLHIV.

**Figure 8:**
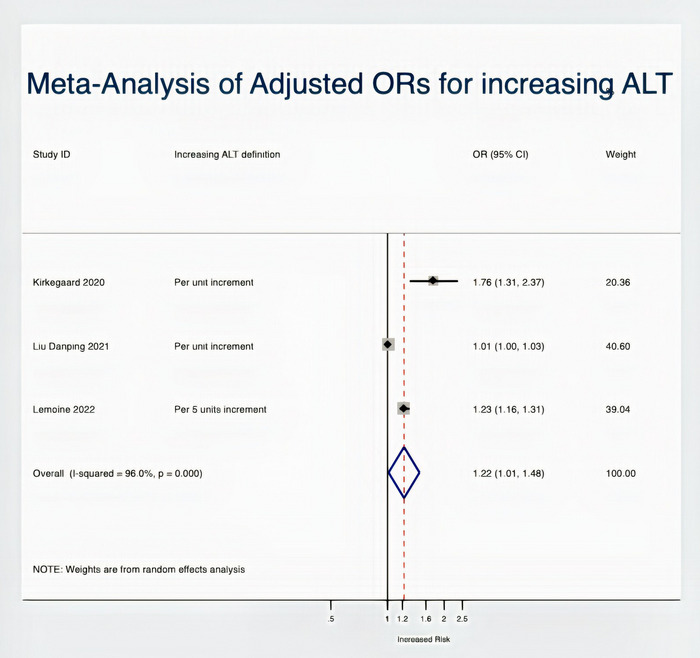
Meta‐analysis of ALT as a risk factor for NAFLD.

Three studies reported the association between alanine transferase (ALT) levels and NAFLD in PLHIV. After pooling the data from these studies, we found that increasing ALT levels were significantly associated with NAFLD risk in PLHIV (Figure 8). Finally, in a random effect model pooling data from four studies, age was not associated with NAFLD (four studies: OR = 1.01; 95% CI: 0.99–1.02; *I* ^2^ = 0%) (Figure S5 in the Supplementary File).

## 4 DISCUSSION

This SR and MA, pooling data from 24 studies published worldwide (*n* = 6326 patients), found a 38% NAFLD and 13% significant fibrosis prevalence in PLHIV. Further, we combined NAFLD risk factor estimates (ORs) and found that increasing triglyceride levels, higher BMI values and dyslipidaemia were associated with higher risk‐adjusted odds of NAFLD among PLHIV.

The NAFLD prevalence estimations did not show clinically relevant/substantial variations in the subgroup analyses by region and income level (Figures 4 and 5); however, most studies were from high‐income/upper‐middle‐income countries, with none from low‐income economies. Of concern, Africa, the world's most affected region by the HIV epidemic [64, 65], was not represented in the MA, as we could not find articles from this continent that fulfilled our inclusion criteria. There is, therefore, an urgent need to address this knowledge gap in future investigations. Furthermore, stakeholders from regions with limited data should consider uniting efforts and resources to determine the NAFLD burden in PLHIV to enhance local patient care [66].

Similar to the prevalence estimates reported herein, a 2017 MA examining NAFLD in 1256 PLHIV found a pooled NAFLD prevalence of 35% with high heterogeneity (*I* ^2^ = 85.3%) [18]. This is similar to NAFLD prevalence in individuals aged 15–49 in the general population, estimated at 34% globally [67]. Although the overall prevalence reported herein (38%) was associated with high heterogeneity (*I* ^2^ = 96.3%), we found that heterogeneity dramatically decreased for VCTE and liver ultrasound when stratifying by diagnostic methods (Figure 6), which means that the high heterogeneity was likely explained (in part) by the variations in NAFLD diagnostic techniques across studies. Notwithstanding the subgroup analyses performed, statistical heterogeneity seems to be an unavoidable issue in MA [68] as true heterogeneity is expected when combining data obtained in different regions, settings and cultures with some degree of variations in clinical practice; however, if SR's inclusion and exclusion criteria apply to the included studies and there are not evident discrepancies between them, a high statistical heterogeneity (*I* ^2^) does not necessarily mean that studies are not combinable and data are inconsistent.

The high NAFLD prevalence in PLHIV is worrying as it depicts a scenario where these individuals are at higher risk of chronic liver disease, including NASH and cirrhosis. Thus, adding an issue to be addressed by the Global Health Sector Strategy on HIV proposed by the World Health Organization [14]. PLHIV stakeholders should undertake efforts to further advance the knowledge about the NAFLD burden in this population, where it is often overlooked. Targeted screening programmes and prevention strategies focusing on PLHIV are needed, as part of comprehensive care provision [16, 69, 70]. Clinical guidelines for PLHV should include fatty liver disease prevention and care, recognizing the importance of early diagnosis and the bidirectional relationship between NAFLD and other metabolic conditions such as diabetes.

We found that dyslipidaemia, increasing triglycerides levels and higher BMI values were associated with higher risk‐adjusted odds of NAFLD in PLHIV and these results do indeed match those observed in earlier studies on the general population [67]. Well‐established risk factors for NAFLD include waist circumference, high triglycerides, diabetes [71] and high BMI [67, 72]. Moreover, it is well‐known that cardiovascular risk factors are highly prevalent in PLHIV [73]. Of particular concern is the clustering of some of these factors forming metabolic syndrome [74, 75], the single most predictive factor of NAFLD. Since BMI is the monitoring method used to track obesity (a hallmark feature of metabolic syndrome), practitioners caring for PLHIV should ensure that patients with a high BMI are supported in attaining a healthier lifestyle, as previous studies have demonstrated that NAFLD prevalence increases linearly with increasing BMI [76, 77], along with the other risks associated with it. For example, higher BMI values have been related to the risk of developing diabetes and a progressively increased risk of complications from diabetes [78].

On the other hand, in the case of dyslipidaemia and high triglyceride levels in PLHIV, its management should be based on their cardiovascular risk and adhere to current guideline recommendations, albeit bearing in mind that high untreated triglyceride levels increase the risk of NAFLD. In contrast, lipid‐lowering agents (i.e. statins) have proven benefits on NAFLD incidence and the progression to hepatic fibrosis in cohorts of NAFLD‐affected individuals [79].

This study also found a prevalence of significant fibrosis of 13% in PLHIV. Although the cut‐off values to define this outcome were lower in the included studies than those proposed in current EASL guidelines [26], we consider this finding relevant for clinical practice as it provides a quantitative estimate of the proportion of “high‐risk” individuals with established NAFLD who merit further medical interventions and closer follow‐up. Previous research found that liver stiffness is significantly higher in PLHIV compared to healthy controls; however, no prior studies comprehensively reported on the prevalence of significant fibrosis in PLHIV. Studies assessing the prevalence of significant liver fibrosis due to NAFLD in the general population are scarce and there is no agreement regarding the most suitable method to estimate the prevalence of fibrosis in the general population. Available data suggest that it is close to 5–6% according to studies from various settings. Using the FIB‐4 score upper cut‐off, the prevalence of advanced fibrosis in a population‐based study in Germany was 1.1% [80]. Whereas in NAFLD patients from the general population, the better‐known risk factor for fibrosis progression is diabetes and other metabolic comorbidities [81, 82], this has not been systematically assessed in most studies on NAFLD in PLHIV. Consequently, we could not analyse the risk factors of significant and/or advanced fibrosis in PLHIV.

The burden of NAFLD and its risk factors among PLHIV described in this review should nourish efforts to create and implement NAFLD screening programmes in this patient population. The fact that the prevalence of NAFLD reported herein was higher than the one reported for the general population is of particular concern.

Since many PLHIV have or will eventually have metabolic syndrome or its components [83], further research is required to uncover the particularities of NAFLD in PLHIV, especially the effect that the time living with the disease may have on the progression and outcomes of the co‐existing hepatic metabolic comorbidity.

### 4.1 Limitations

This study has limitations and results should be interpreted in the context of the study design. First, the review was not registered in PROSPERO. Second, the main limitation of the present MA is the high heterogeneity found across studies, which could theoretically comprise the validity of the pooled estimates presented. Third, the used‐cut‐offs for CAP and liver stiffness have been redefined over time according to recent EASL guidelines [26] and this affects the outcomes of interest synthesized in this review. However, as the MAs were based on methodologically similar studies with analogous inclusion and exclusion criteria, one can infer that clinical and methodological heterogeneity levels were likely low. Moreover, previous simulation studies demonstrated that determining levels of heterogeneity is of little value at the extremes of it [84], such as in this study.

Fourth, the sources from which the pooled prevalence estimates were calculated are subjected to selection bias as they represent the setting where they were recruited, albeit not the whole PLHIV population. Thus, there is potential for meta‐bias and overestimation because the meta‐analytic pooled estimates may not accurately represent the true NAFLD prevalence in the whole PLHIV population in the society. Nevertheless, we consider the results from the present study reliable because meta‐analytic methods may provide a more precise estimate of such measures than any individual study contributing to the pooled analysis (MA).

Fifth, due to a lack of data in the analysed studies, we could not establish the effect of the duration of HIV and antiretroviral therapy on NAFLD. Therefore, additional studies should address this and NAFLD's natural history and focus on the highly prevalent burden of comorbidities with particular attention to finding a differential effect of these factors in PLHIV compared to the general population.

Finally, the study was limited by the lack of information from low‐income economies, making these findings not generalizable to a significant share of the world's population residing in these often‐underserved regions.

## 5 CONCLUSIONS

The burden of NAFLD and significant fibrosis in PLHIV is significant. Therefore, targeted efforts to screen and diagnose NAFLD in this population are needed. Health services for PLHIV could include ways to target NAFLD risk factors, screen for liver disease and implement interventions to treat those with significant fibrosis or more advanced stages of liver disease. Taking no action to screen for NAFLD in PLHIV and address this often‐overlooked metabolic comorbidity should not be an option.

## Authors’ Contributions

Conceptualization and design: JRE, RMN and JMP. Data collection: JRE, RMN and ES. Drafting of the first manuscript: RMN, JRE, ES and JMP. Data analyses: RMN. Data interpretation: JRE, RMN, JN, JVL, JMS, AC and JMP. Critical revision of the manuscript: JN, JVL, JMS and AC. Supervision: JMP. Access and verification of data: JMP. All authors confirm that they had full access to all the data in the study and accept responsibility to submit for publication.

## COMPETING INTERESTS

JVL acknowledges grants and speaker fees from AbbVie, Gilead Sciences and MSD and speaker fees from Genfit, Intercept, Jannsen and ViiV, outside of the submitted work. JMP reports having received consulting fees from Boehringer Ingelheim and Novo Nordisk. He has received speaking fees from Gilead, and travel expenses from Gilead, Rubió, Pfizer, Astellas, MSD, CUBICIN and Novo Nordisk. He has received educational and research support from Gilead, Pfizer, Astellas, Accelerate, Novartis, Abbvie, ViiV and MSD, and funds from European Commission/EFPIA IMI2 853966‐2, IMI2 777377, H2020 847989 and ISCIII PI19/01898 (all outside the submitted work). JVL acknowledges support from the Spanish Ministry of Science and Innovation and State Research Agency through the “Centro de Excelencia Severo Ochoa 2019–2023” Program (CEX2018‐000806‐S), and support from the Generalitat de Catalunya through the CERCA Program.

JMS reports consultancy for Apollo Endosurgery, Albireo Pharma Inc, Bayer, BMS, Boehringer Ingelheim, Echosens, Genfit, Gilead Sciences, GSK, Heel GmbH, Intercept Pharmaceuticals, Ipsen, Inventiva Pharma, Julius Clinical, Madrigal, MSD, Nordic Bioscience, Novartis, Novo Nordisk, Pfizer, Roche, Sanofi, Shinogi, Siemens Healthcare GmbH, Summit Clinical Research; research funding from Gilead Sciences, Boehringer Ingelheim, Nordic Bioscience, Siemens Healthcare GmbH and speaker honoraria from MedPublico GmbH, Boehringer Ingelheim (all outside the submitted work).

## Funding

The author(s) received no financial support for the research, authorship and/or publication of this article.

## Supporting information

Supporting Information

## Data Availability

The datasets and Stata commands generated and used to perform the present systematic review and meta‐analysis can be available from the corresponding author at reasonable request.

## References

[1] Younossi ZM , Koenig AB , Abdelatif D , Fazel Y , Henry L , Wymer M . Global epidemiology of nonalcoholic fatty liver disease—meta‐analytic assessment of prevalence, incidence, and outcomes. Hepatology. 2016;64(1):73–84. 26707365 10.1002/hep.28431

[2] Riazi K , Azhari H , Charette JH , Underwood FE , King JA , Afshar EE , et al. The prevalence and incidence of NAFLD worldwide: a systematic review and meta‐analysis. Lancet Gastroenterol Hepatol. 2022;7(9):851–61. 35798021 10.1016/S2468-1253(22)00165-0

[3] Ekstedt M , Nasr P , Kechagias S . Natural history of NAFLD/NASH. Curr Hepatol Rep. 2017;16(4):391–7. 29984130 10.1007/s11901-017-0378-2 PMC6022523

[4] Younossi Z , Anstee QM , Marietti M , Hardy T , Henry L , Eslam M , et al. Global burden of NAFLD and NASH: trends, predictions, risk factors and prevention. Nat Rev Gastroenterol Hepatol. 2018;15(1):11–20. 28930295 10.1038/nrgastro.2017.109

[5] Estes C , Razavi H , Loomba R , Younossi Z , Sanyal AJ . Modeling the epidemic of nonalcoholic fatty liver disease demonstrates an exponential increase in burden of disease. Hepatology. 2018;67(1):123–33. 28802062 10.1002/hep.29466 PMC5767767

[6] Araújo AR , Rosso N , Bedogni G , Tiribelli C , Bellentani S . Global epidemiology of non‐alcoholic fatty liver disease/non‐alcoholic steatohepatitis: what we need in the future. Liver Int. 2018;38(Suppl 1):47–51. 29427488 10.1111/liv.13643

[7] Kanwal F , Kramer JR , Mapakshi S , Natarajan Y , Chayanupatkul M , Richardson PA , et al. Risk of hepatocellular cancer in patients with non‐alcoholic fatty liver disease. Gastroenterology. 2018;155(6):1828–37.e2. 30144434 10.1053/j.gastro.2018.08.024 PMC6279617

[8] The global, regional, and national burden of cirrhosis by cause in 195 countries and territories, 1990–2017: a systematic analysis for the Global Burden of Disease Study 2017. Lancet Gastroenterol Hepatol. 2020;5(3):245–66. 31981519 10.1016/S2468-1253(19)30349-8 PMC7026710

[9] Younossi ZM , Blissett D , Blissett R , Henry L , Stepanova M , Younossi Y , et al. The economic and clinical burden of nonalcoholic fatty liver disease in the United States and Europe. Hepatology. 2016;64(5):1577–86. 27543837 10.1002/hep.28785

[10] Younossi ZM , Yilmaz Y , Yu ML , Wai‐Sun Wong V , Fernandez MC , Isakov VA , et al. Clinical and patient‐reported outcomes from patients with nonalcoholic fatty liver disease across the world: data from the Global Non‐Alcoholic Steatohepatitis (NASH)/Non‐Alcoholic Fatty Liver Disease (NAFLD) Registry. Clin Gastroenterol Hepatol. 2022;20(10):2296–306.e6. 34768009 10.1016/j.cgh.2021.11.004

[11] Schattenberg JM , Lazarus JV , Newsome PN , Serfaty L , Aghemo A , Augustin S , et al. Disease burden and economic impact of diagnosed non‐alcoholic steatohepatitis in five European countries in 2018: a cost‐of‐illness analysis. Liver Int. 2021;41(6):1227–42. 33590598 10.1111/liv.14825 PMC8252761

[12] Smith CJ , Ryom L , Weber R , Morlat P , Pradier C , Reiss P , et al. Trends in underlying causes of death in people with HIV from 1999 to 2011 (D:A:D): a multicohort collaboration. Lancet. 2014;384(9939):241–8. 25042234 10.1016/S0140-6736(14)60604-8

[13] Lake JE , Overton T , Naggie S , Sulkowski M , Loomba R , Kleiner DE , et al. Expert panel review on nonalcoholic fatty liver disease in persons with human immunodeficiency virus. Clin Gastroenterol Hepatol. 2022;20(2):256–68. 33069882 10.1016/j.cgh.2020.10.018 PMC9069630

[14] Lazarus JV , Safreed‐Harmon K , Barton SE , Costagliola D , Dedes N , del Amo Valero J , et al. Beyond viral suppression of HIV – the new quality of life frontier. BMC Med. 2016;14(1):94. 27334606 10.1186/s12916-016-0640-4 PMC4916540

[15] Kall M , Marcellin F , Harding R , Lazarus JV , Carrieri P . Patient‐reported outcomes to enhance person‐centred HIV care. Lancet HIV. 2020;7(1):e59–68. 31776101 10.1016/S2352-3018(19)30345-5

[16] Lazarus JV , Safreed‐Harmon K , Kamarulzaman A , Anderson J , Leite RB , Behrens G , et al. Consensus statement on the role of health systems in advancing the long‐term well‐being of people living with HIV. Nat Commun. 2021;12(1):4450. 34272399 10.1038/s41467-021-24673-w PMC8285468

[17] WHO . Global health sector strategies on, respectively, HIV, viral hepatitis and sexually transmitted infections for the period 2022–2030 [Internet]. 2022. Available from: https://www.who.int/publications/i/item/9789240053779 Accessed Jan 5, 2022.

[18] Maurice JB , Patel A , Scott AJ , Patel K , Thursz M , Lemoine M . Prevalence and risk factors of nonalcoholic fatty liver disease in HIV‐monoinfection. AIDS. 2017;31(11):1621–32. 28398960 10.1097/QAD.0000000000001504

[19] Barendregt JJ , Doi SA , Lee YY , Norman RE , Vos T . Meta‐analysis of prevalence. J Epidemiol Community Health. 2013;67(11):974–8. 23963506 10.1136/jech-2013-203104

[20] Nyaga VN , Arbyn M , Aerts M . Metaprop: a Stata command to perform meta‐analysis of binomial data. Arch Public Health. 2014;72(1):39. 25810908 10.1186/2049-3258-72-39 PMC4373114

[21] Singh P , Arora A , Strand TA , Leffler DA , Catassi C , Green PH , et al. Global prevalence of celiac disease: systematic review and meta‐analysis. Clin Gastroenterol Hepatol. 2018;16(6):823–36.e2. 29551598 10.1016/j.cgh.2017.06.037

[22] Ismail Z , Elbayoumi H , Fischer CE , Hogan DB , Millikin CP , Schweizer T , et al. Prevalence of depression in patients with mild cognitive impairment: a systematic review and meta‐analysis. JAMA Psychiatry. 2017;74(1):58–67. 27893026 10.1001/jamapsychiatry.2016.3162

[23] Bonyadi P , Saleh NT , Dehghani M , Yamini M , Amini K . Prevalence of antibiotic resistance of *Pseudomonas aeruginosa* in cystic fibrosis infection: a systematic review and meta‐analysis. Microb Pathog. 2022;165:105461. 35240288 10.1016/j.micpath.2022.105461

[24] Higgins JP , Thomas J , Chandler J , Cumpston M , Li T , Page MJ , et al. Cochrane handbook for systematic reviews of interventions. 2nd edition. Chichester: John Wiley & Sons; 2019.

[25] Moher D , Liberati A , Tetzlaff J , Altman DG . Preferred Reporting Items for Systematic Reviews and Meta‐Analyses: the PRISMA statement. J Clin Epidemiol. 2009;62(10):1006–12. 19631508 10.1016/j.jclinepi.2009.06.005

[26] EASL . EASL Clinical Practice Guidelines on non‐invasive tests for evaluation of liver disease severity and prognosis ‐ 2021 update. J Hepatol. 2021;75(3):659–89. 34166721 10.1016/j.jhep.2021.05.025

[27] Robinson KA , Dickersin K . Development of a highly sensitive search strategy for the retrieval of reports of controlled trials using PubMed. Int J Epidemiol. 2002;31(1):150–3. 11914311 10.1093/ije/31.1.150

[28] Biondi‐Zoccai GGL , Agostoni P , Abbate A , Testa L , Burzotta F . A simple hint to improve Robinson and Dickersin's highly sensitive PubMed search strategy for controlled clinical trials. Int J Epidemiol. 2005;34(1):224–5. 15659479 10.1093/ije/dyh311

[29] Aromataris E , Riitano D . Constructing a search strategy and searching for evidence. A guide to the literature search for a systematic review. Am J Nurs. 2014;114(5):49–56. 10.1097/01.NAJ.0000446779.99522.f6 24759479

[30] Ouzzani M , Hammady H , Fedorowicz Z , Elmagarmid A . Rayyan—a web and mobile app for systematic reviews. Syst Rev. 2016;5(1):210. 27919275 10.1186/s13643-016-0384-4 PMC5139140

[31] Aromataris E , Stern C , Lockwood C , Barker TH , Klugar M , Jadotte Y , et al. JBI series paper 2: tailored evidence synthesis approaches are required to answer diverse questions: a pragmatic evidence synthesis toolkit from JBI. J Clin Epidemiol. 2022;150:196–202. 35429608 10.1016/j.jclinepi.2022.04.006

[32] Aromataris E , Munn Z . JBI manual for evidence synthesis. 2020.

[33] Zeng X , Zhang Y , Kwong JSW , Zhang C , Li S , Sun F , et al. The methodological quality assessment tools for preclinical and clinical studies, systematic review and meta‐analysis, and clinical practice guideline: a systematic review. J Evid Based Med. 2015;8(1):2–10. 25594108 10.1111/jebm.12141

[34] Porritt K , Gomersall J , Lockwood C . JBI's Systematic Reviews: study selection and critical appraisal. Am J Nurs. 2014;114(6):47–52. 10.1097/01.NAJ.0000450430.97383.64 24869584

[35] Hunter JP , Saratzis A , Sutton AJ , Boucher RH , Sayers RD , Bown MJ . In meta‐analyses of proportion studies, funnel plots were found to be an inaccurate method of assessing publication bias. J Clin Epidemiol. 2014;67(8):897–903. 24794697 10.1016/j.jclinepi.2014.03.003

[36] Harris R , Bradburn M , Deeks J , Harbord R , Altman D , Sterne J . Metan: fixed‐ and random‐effects meta‐analysis. Stata J. 2008;8(1):3–28.

[37] Freeman MF , Tukey JW . Transformations related to the angular and the square root. Ann Math Stat. 1950;21(4):607–11.

[38] Newcombe RG . Two‐sided confidence intervals for the single proportion: comparison of seven methods. Stat Med. 1998;17(8):857–72. 9595616 10.1002/(sici)1097-0258(19980430)17:8<857::aid-sim777>3.0.co;2-e

[39] DerSimonian R , Laird N . Meta‐analysis in clinical trials. Control Clin Trials. 1986;7(3):177–88. 3802833 10.1016/0197-2456(86)90046-2

[40] De A , Duseja A , Badhala P , Taneja S , Sharma A , Arora S . Indian patients with human immunodeficiency virus infection have high prevalence but mild severity of non‐alcoholic fatty liver disease. Diabetes Metab Syndr Clin Res Rev. 2022;16(12):102679. 10.1016/j.dsx.2022.102679 36450180

[41] Busca C , Sánchez‐Conde M , Rico M , Rosas M , Valencia E , Moreno A , et al. Assessment of noninvasive markers of steatosis and liver fibrosis in human immunodeficiency virus‐monoinfected patients on stable antiretroviral regimens. Open Forum Infect Dis. 2022;9(7):ofac279. 35873289 10.1093/ofid/ofac279 PMC9297309

[42] Bischoff J , Gu W , Schwarze‐Zander C , Boesecke C , Wasmuth JC , van Bremen K , et al. Stratifying the risk of NAFLD in patients with HIV under combination antiretroviral therapy (cART). eClinicalMedicine. 2021;40:1–11. 10.1016/j.eclinm.2021.101116 PMC8427211 34522873

[43] Liu D , Shen Y , Zhang R , Xun J , Wang J , Liu L , et al. Prevalence and risk factors of metabolic associated fatty liver disease among people living with HIV in China. J Gastroenterol Hepatol. 2021;36(6):1670–8. 33140878 10.1111/jgh.15320

[44] Pezzini MF , Cheinquer H , de Araujo A , Schmidt‐Cerski CT , Sprinz E , Herz‐Wolff F , et al. Hepatic steatosis among people living with HIV in Southern Brazil: prevalence and risk factors. Sci Rep. 2020;10(1):1–6. 32427918 10.1038/s41598-020-65133-7 PMC7237667

[45] de Almeida CF , da Silva PS , de Cardoso CS A , Moreira NG , Antunes JC , de Andrade MM , et al. Relationship between dietary fatty acid intake with nonalcoholic fatty liver disease and liver fibrosis in people with HIV. Nutrients. 2021;13(10):1–17. 10.3390/nu13103462 PMC8539489 34684463

[46] Lui G , Wong V , Wong G , Chu W , Wong CKK , Yung I , et al. Liver fibrosis and fatty liver in Asian HIV‐infected patients. Aliment Pharmacol Ther. 2016;44(4):411–21. 27301337 10.1111/apt.13702

[47] Jongraksak T , Sobhonslidsuk A , Jatchavala J , Warodomwichit D , Kaewduang P , Sungkanuparph S . Prevalence and predicting factors of metabolic‐associated fatty liver disease diagnosed by transient elastography with controlled attenuation parameters in HIV‐positive people. Int J STD AIDS. 2021;32(3):266–75. 33334267 10.1177/0956462420960997

[48] Kaplan A , Simon TG , Henson JB , Wang T , Zheng H , Osganian SA , et al. Relationship between nonalcoholic fatty liver disease and cardiovascular disease in persons with HIV. J Acquir Immune Defic Syndr. 2020;84(4):400–4. 32235172 10.1097/QAI.0000000000002359 PMC10462389

[49] Lallukka‐Brück S , Isokuortti E , Luukkonen PK , Hakkarainen A , Lundbom N , Sutinen J , et al. Natural course of nonalcoholic fatty liver disease and type 2 diabetes in patients with human immunodeficiency virus with and without combination antiretroviral therapy‐associated lipodystrophy: a 16‐year follow‐up study. Clin Infect Dis. 2020;70(8):1708–16. 31131845 10.1093/cid/ciz435

[50] Lombardi R , Lever R , Smith C , Marshall N , Rodger A , Bhagani S , et al. Liver test abnormalities in patients with HIV mono‐infection: assessment with simple noninvasive fibrosis markers. Ann Gastroenterol. 2017;30(3):349–56. 28469366 10.20524/aog.2017.0141 PMC5411386

[51] Michel M , Labenz C , Anders M , Wahl A , Girolstein L , Kaps L , et al. Effect of hepatic steatosis and associated metabolic comorbidities on health‐related quality of life in people living with HIV. Hepatol Commun. 2022;6(8):2011–21. 35411570 10.1002/hep4.1958 PMC9315116

[52] Crum‐Cianflone N , Dilay A , Collins G , Asher D , Campin R , Medina S , et al. Nonalcoholic fatty liver disease among HIV‐infected persons. J Acquir Immune Defic Syndr. 2009;50(5):464–73. 19225402 10.1097/QAI.0b013e318198a88a PMC2782375

[53] Vujanović M , Brkić‐Jovanović N , Ilić D , Drvendžija Z , Srdić‐Galić B , Turkulov V , et al. Associations of visceral fat thickness and anthropometric measurements with non‐alcoholic fatty liver disease development in male patients mono‐infected with human immunodeficiency virus. South Afr J HIV Med. 2019;20(1):968. 31534788 10.4102/sajhivmed.v20i1.968 PMC6739542

[54] Nishijima T , Gatanaga H , Shimbo T , Komatsu H , Nozaki Y , Nagata N , et al. Traditional but not HIV‐related factors are associated with nonalcoholic fatty liver disease in Asian patients with HIV‐1 infection. PLoS One. 2014;9(1): e87596. 10.1371/journal.pone.0087596 PMC3909216 24498148

[55] Rasoulinejad M , Seyed Alinaghi SA , Sohrabi MR , Badie BM , Manshadi SAD , Nezhad MH , et al. Prevalence and factors associated with hepatic steatosis and fibrosis using fibroscan in HIV‐positive patients treated with anti‐retroviral (ARV) medicines referred to the biggest hospital in Tehran, 2018 to 2019. Open AIDS J. 2020;14(1):108–13.

[56] Sebastiani G , Milic J , Cervo A , Saeed S , Krahn T , Kablawi D , et al. Two‐tier care pathways for liver fibrosis associated to non‐alcoholic fatty liver disease in HIV mono‐infected patients. J Pers Med. 2022;12(2):282. 35207770 10.3390/jpm12020282 PMC8874585

[57] Kardashian A , Ma Y , Scherzer R , Price JC , Sarkar M , Korn N , et al. Sex differences in the association of HIV infection with hepatic steatosis. AIDS. 2017;31(3):365–73. 27831949 10.1097/QAD.0000000000001334 PMC5233556

[58] Kirkegaard‐Klitbo DM , Fuchs A , Stender S , Sigvardsen PE , Kühl JT , Kofoed KF , et al. Prevalence and risk factors of moderate‐to‐severe hepatic steatosis in human immunodeficiency virus infection: the Copenhagen Co‐morbidity Liver Study. J Infect Dis. 2020;222(8):1353–62. 32417886 10.1093/infdis/jiaa246

[59] Lemoine M , Assoumou L , Girard PM , Valantin MA , Katlama C , De Wit S , et al. Screening HIV patients at risk for NAFLD using MRI‐PDFF and transient elastography: a European Multicenter Prospective Study. Clin Gastroenterol Hepatol. 2022;21(3):713–722. 10.1016/j.cgh.2022.03.048 35436624

[60] Price JC , Wang R , Seaberg EC , Budoff MJ , Kingsley LA , Palella FJ , et al. The association of inflammatory markers with nonalcoholic fatty liver disease differs by human immunodeficiency virus serostatus. Open Forum Infect Dis. 2017;4(3):ofx153. 28929125 10.1093/ofid/ofx153 PMC5601080

[61] Sterling RK , Smith PG , Brunt EM . Hepatic steatosis in human immunodeficiency virus: a prospective study in patients without viral hepatitis, diabetes, or alcohol abuse. J Clin Gastroenterol. 2013;47(2):182–7. 23059409 10.1097/MCG.0b013e318264181d PMC3544978

[62] Aepfelbacher JA , Balmaceda J , Purdy J , Mattingly A , Zambell K , Hawkins K , et al. Increased prevalence of hepatic steatosis in young adults with lifelong HIV. J Infect Dis. 2019;220(2):266–9. 30852587 10.1093/infdis/jiz096 PMC6581896

[63] Ajmera VH , Cachay ER , Ramers CB , Bassirian S , Singh S , Bettencourt R , et al. Optimal threshold of controlled attenuation parameter for detection of HIV‐associated NAFLD with magnetic resonance imaging as the reference standard. Clin Infect Dis. 2021;72(12):2124–31. 32975278 10.1093/cid/ciaa429 PMC8204791

[64] Dwyer‐Lindgren L , Cork MA , Sligar A , Steuben KM , Wilson KF , Provost NR , et al. Mapping HIV prevalence in sub‐Saharan Africa between 2000 and 2017. Nature. 2019;570(7760):189–93. 31092927 10.1038/s41586-019-1200-9 PMC6601349

[65] Jahagirdar D , Walters MK , Novotney A , Brewer ED , Frank TD , Carter A , et al. Global, regional, and national sex‐specific burden and control of the HIV epidemic, 1990–2019, for 204 countries and territories: the Global Burden of Diseases Study 2019. Lancet HIV. 2021;8(10):e633–51. 34592142 10.1016/S2352-3018(21)00152-1 PMC8491452

[66] Safreed‐Harmon K , Anderson J , Azzopardi‐Muscat N , Behrens GMN , d'Arminio Monforte A , Davidovich U , et al. Reorienting health systems to care for people with HIV beyond viral suppression. Lancet HIV. 2019;6(12):e869–77. 31776099 10.1016/S2352-3018(19)30334-0

[67] Ge X , Zheng L , Wang M , Du Y , Jiang J . Prevalence trends in non‐alcoholic fatty liver disease at the global, regional and national levels, 1990–2017: a population‐based observational study. BMJ Open. 2020;10(8):e036663. 10.1136/bmjopen-2019-036663 PMC7402189 32747349

[68] Higgins JPT . Commentary: heterogeneity in meta‐analysis should be expected and appropriately quantified. Int J Epidemiol. 2008;37(5):1158–60. 18832388 10.1093/ije/dyn204

[69] Kaps L , Labenz C , Galle PR , Weinmann‐Menke J , Kostev K , Schattenberg JM . Non‐alcoholic fatty liver disease increases the risk of incident chronic kidney disease. United Eur Gastroenterol J. 2020;8(8):942–8. 10.1177/2050640620944098 PMC7707877 32698692

[70] Lazarus JV , Mark HE , Anstee QM , Arab JP , Batterham RL , Castera L , et al. Advancing the global public health agenda for NAFLD: a consensus statement. Nat Rev Gastroenterol Hepatol. 2022;19(1):60–78. 34707258 10.1038/s41575-021-00523-4

[71] Cusi K , Sanyal AJ , Zhang S , Hartman ML , Bue‐Valleskey JM , Hoogwerf BJ , et al. Non‐alcoholic fatty liver disease (NAFLD) prevalence and its metabolic associations in patients with type 1 diabetes and type 2 diabetes. Diabetes Obes Metab. 2017;19(11):1630–4. 28417532 10.1111/dom.12973

[72] Lu FB , Hu ED , Xu LM , Chen L , Wu JL , Li H , et al. The relationship between obesity and the severity of non‐alcoholic fatty liver disease: systematic review and meta‐analysis. Expert Rev Gastroenterol Hepatol. 2018;12(5):491–502. 29609501 10.1080/17474124.2018.1460202

[73] Knobel H , Domingo P , Suarez‐Lozano I , Gutierrez F , Estrada V , Palacios R , et al. Rate of cardiovascular, renal and bone disease and their major risks factors in HIV‐infected individuals on antiretroviral therapy in Spain. Enfermedades Infecc y Microbiol Clin (English ed). 2019;37(6):373–9. 10.1016/j.eimc.2018.09.015 30389268

[74] Kim D , Touros A , Kim WR . Nonalcoholic fatty liver disease and metabolic syndrome. Clin Liver Dis. 2018;22(1):133–40. 29128053 10.1016/j.cld.2017.08.010

[75] Sookoian S , Pirola CJ . Systematic review with meta‐analysis: risk factors for non‐alcoholic fatty liver disease suggest a shared altered metabolic and cardiovascular profile between lean and obese patients. Aliment Pharmacol Ther. 2017;46(2):85–95. 28464369 10.1111/apt.14112

[76] Duseja A , Chalasani N . Epidemiology and risk factors of nonalcoholic fatty liver disease (NAFLD). Hepatol Int. 2013;7(Suppl 2):755–64. 26202291 10.1007/s12072-013-9480-x

[77] Eguchi Y , Hyogo H , Ono M , Mizuta T , Ono N , Fujimoto K , et al. Prevalence and associated metabolic factors of nonalcoholic fatty liver disease in the general population from 2009 to 2010 in Japan: a multicenter large retrospective study. J Gastroenterol. 2012;47(5):586–95. 22328022 10.1007/s00535-012-0533-z

[78] Gray N , Picone G , Sloan F , Yashkin A . Relation between BMI and diabetes mellitus and its complications among US older adults. South Med J. 2015;108(1):29–36. 25580754 10.14423/SMJ.0000000000000214 PMC4457375

[79] Tzanaki I , Agouridis AP , Kostapanos MS . Is there a role of lipid‐lowering therapies in the management of fatty liver disease? World J Hepatol. 2022;14(1):119–39. 35126843 10.4254/wjh.v14.i1.119 PMC8790403

[80] Huber Y , Schulz A , Schmidtmann I , Beutel M , Pfeiffer N , Münzel T , et al. Prevalence and risk factors of advanced liver fibrosis in a population‐based study in Germany. Hepatol Commun. 2022;6(6):1457–66. 35122404 10.1002/hep4.1899 PMC9134815

[81] Pais R , Charlotte F , Fedchuk L , Bedossa P , Lebray P , Poynard T , et al. A systematic review of follow‐up biopsies reveals disease progression in patients with non‐alcoholic fatty liver. J Hepatol. 2013;59(3):550–6. 23665288 10.1016/j.jhep.2013.04.027

[82] Schuppan D , Surabattula R , Wang XY . Determinants of fibrosis progression and regression in NASH. J Hepatol. 2018;68(2):238–50. 29154966 10.1016/j.jhep.2017.11.012

[83] Smit M , Brinkman K , Geerlings S , Smit C , Thyagarajan K , van SA , et al. Future challenges for clinical care of an ageing population infected with HIV: a modelling study. Lancet Infect Dis. 2015;15(7):810–8. 26070969 10.1016/S1473-3099(15)00056-0 PMC4528076

[84] Melsen WG , Bootsma MCJ , Rovers MM , Bonten MJM . The effects of clinical and statistical heterogeneity on the predictive values of results from meta‐analyses. Clin Microbiol Infect. 2014;20(2):123–9. 24320992 10.1111/1469-0691.12494

