# Supplementary material for: Uncovering the NAFLD burden in people living with HIV from high‐ and middle‐income nations: a meta‐analysis with a data gap from Subsaharan Africa
Source: J Int AIDS Soc. 2023 Mar 16;26(3):e26072. doi: 10.1002/jia2.26072 (PMC10018385; doi:10.1002/jia2.26072)

## Supplementary File 1:

The present electronic supplementary material was prepared for the manuscript ***“A meta- analysis of the prevalence and risk factors of non-alcoholic fatty liver disease in people living with HIV”.*** For questions about the information herein contained, please get in touch with the corresponding author:

Juan M. Pericas MD, MPH , PhD

e-mail address: [juanmanuel.pericas@vallhebron.cat](mailto:juanmanuel.pericas@vallhebron.cat), [juan.pericas@vhir.org](mailto:juan.pericas@vhir.org) Liver Unit

Internal Medicine Department Vall d’Hebron University Hospital Barcelona, Spain

## This supplement contains the following items:

### Search Strategies

1. ***Risk of Bias within studies - The quality of the studies was evaluated using the JBI’s Critical Appraisal Tools for Cross-Sectional, Case-control, and Cohort studies (***[***https://jbi.global/critical-appraisal-tools***](https://jbi.global/critical-appraisal-tools)***):***

### Figure S1. Methodological quality assessment of Cross-sectional studies Figure S2. Methodological quality assessment of Case-Control studies Figure S3. Methodological quality assessment of Cohort studies

1. ***Stata Statistical Commands used to perform the meta-analyses.***

### Table S1. Studies’ inclusion and exclusion criteria

1. ***Table S2. Characteristics and reasons for exclusion of key studies that were not included in the systematic review.***

### Figure S4. Subgroup analysis by type of study design (for the meta-analysis of prevalence)

1. ***Figure S5. Meta-analysis of adjusted ORs of the association between Age and NAFLD in PLHIV***

# Electronic search Strategies:

## MEDLINE (through OVID): 217

1. exp HIV/
2. exp Acquired Immunodeficiency Syndrome/
3. exp HIV-1/
4. exp HIV-2/
5. human immunodeficiency virus.mp.
6. exp Fatty Liver/
7. exp Non-alcoholic Fatty Liver Disease/
8. hepatic steatosis.mp.
9. NAFL.mp.
10. NAFLD.mp.
11. NASH.mp.
12. non-alcoholic steatohepatitis.mp.
13. MAFLD
14. 1 or 2 or 3 or 4 or 5
15. 6 or 7 or 8 or 9 or 10 or 11 or 12 or 13

15. 14 and 15

## Scopus: 1601

( TITLE-ABS-KEY ( {HIV infection} OR {HIV} OR {HIV 1} OR {HIV 2} OR {human

immunodeficiency virus} ) AND TITLE-ABS-KEY ( "fatty liver" OR {NAFLD} OR {non alcoholic fatty liver disease} OR {NASH} OR {non alcoholic steatohepatitis} ) )

# Evaluation of the risk of bias:

The Joanna Briggs Institute (JBI) has developed critical appraisal tools to assess the methodological quality of research studies and identify potential biases in design, conduct, and analysis (<https://jbi.global/critical-appraisal-tools>). These tools are intended to be utilized during the systematic review process, where all studies selected for inclusion must undergo a rigorous appraisal. The JBI critical appraisal tools have undergone extensive peer review and have been approved by the JBI Scientific Committee, thus ensuring their reliability and validity for research evaluations.

The JBI’s tools are designed to evaluate the quality of each study across several methodological domains specific to the study design. This systematic review used the tools/checklists for cross-sectional, case-control, and cohort studies. Each item is evaluated as yes/present, no/not present, unclear, and not applicable. We represented these responses in a color plot where green (yes), yellow (unclear), red (no), and grey (not applicable) corresponded to the categories mentioned.

## Figure S1. Methodological quality assessment of Cross-sectional studies Figure S2. Methodological quality assessment of Case-Control studies Figure S3. Methodological quality assessment of Cohort studies

**Figure S1. Methodological quality assessment of Cross-sectional studies**

**Answers:** Yes (green-low risk of bias), No (red-high risk of bias), unclear (yellow – unclear risk of bias), not applicable (gray – domain not applicable for the study)


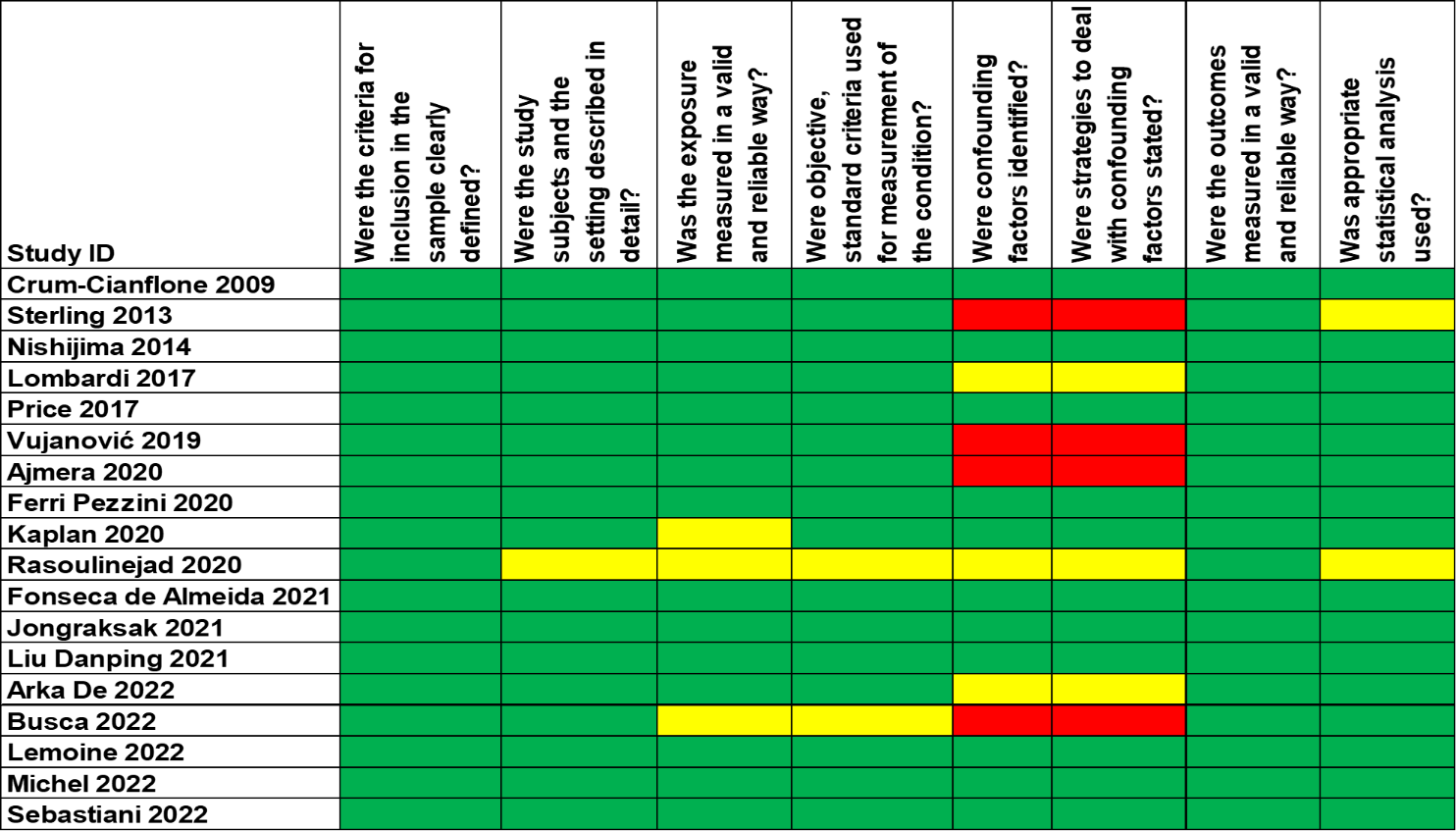


## Figure S2. Methodological quality assessment of Case-Control studies

**Answers:** Yes (green-low risk of bias), No (red-high risk of bias), unclear (yellow – unclear risk of bias), not applicable (gray – domain not applicable for the study)
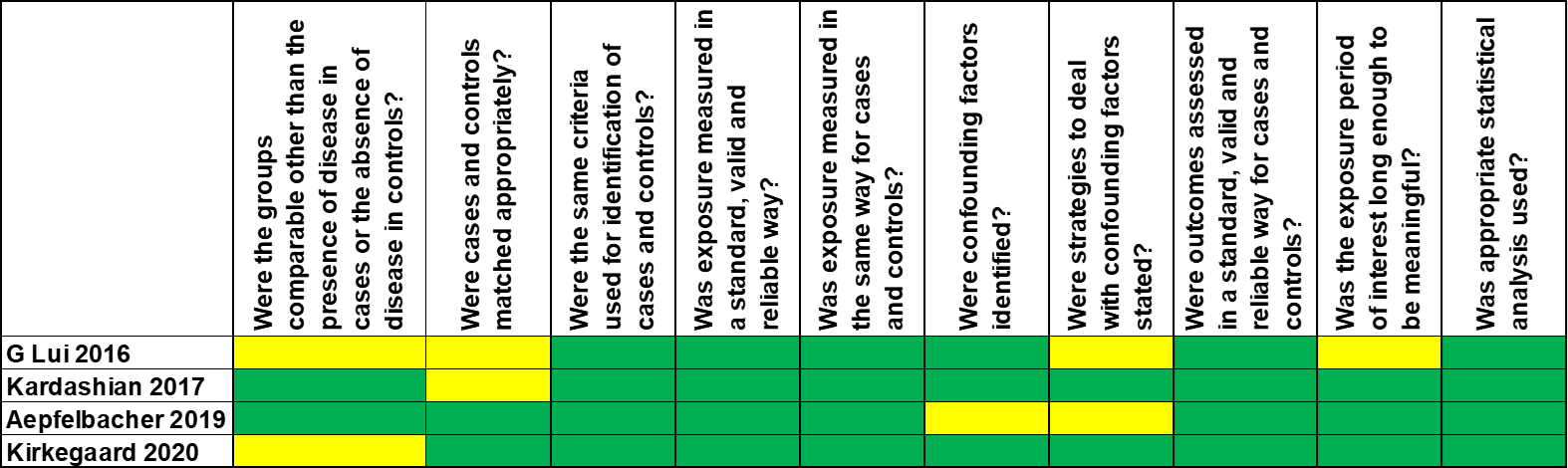


## Figure S3. Methodological quality assessment of Cohort studies

**Answers:** Yes (green-low risk of bias), No (red-high risk of bias), unclear (yellow – unclear risk of bias), not applicable (gray – domain not applicable for the study)


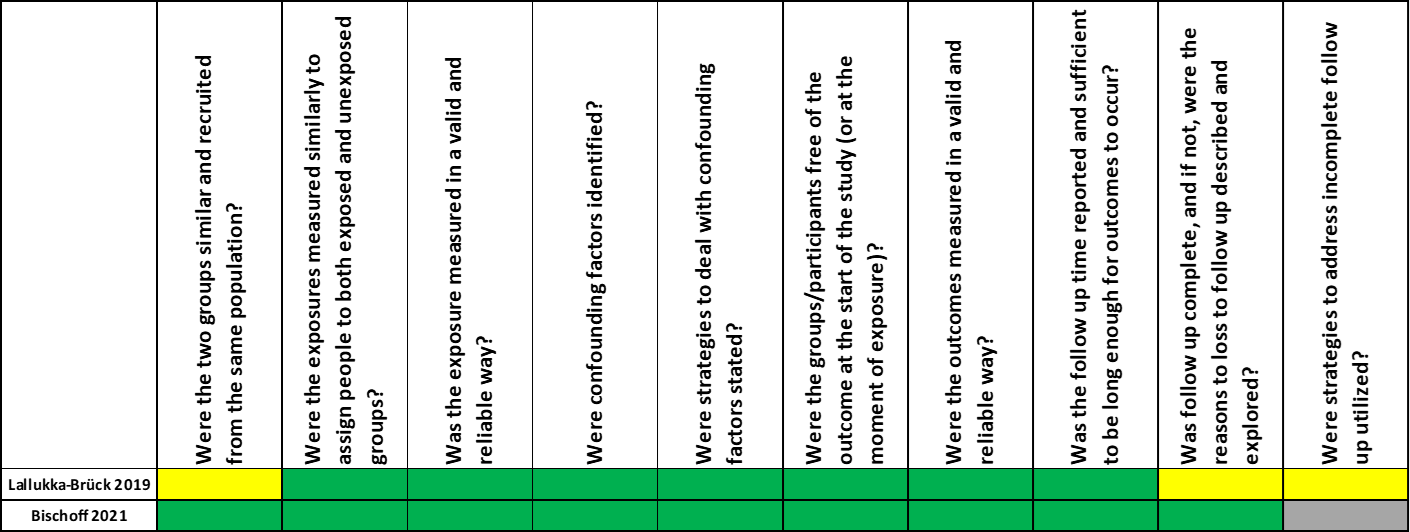


# Stata Commands used to perform the meta-analyses.

A detailed explanation of the analyses is available in the methods section of the manuscript; here, we provide the commands as were executed in the Stata statistical program

### Commands for the meta-analysis of prevalence

For the meta-analyses of prevalence, we used the “metaprop” command in Stata. For this command, we required the number of patients with the outcome of interest (n_NAFLD) and the total number of patients (N_PLHIV): n/N. With these data, we executed the following commands:

metaprop n_NAFLD N_PLHIV, random ftt cimethod(score)

For subgroup analyses we used the option “by”:

metaprop n_NAFLD N_PLHIV, by(subgroup) random ftt cimethod(score)

### Commands for the meta-analysis of risk factors

We used the “metan” command in Stata for meta-analyses of risk factors. For this command, we required the effect estimate (adjusted OR), the lower limit (lci), and the upper limit (uci) of the OR 95% confidence interval. It is necessary to log-transform the ORs and the 95%. The log-transformed values are used as inputs to the metan command, and then using the “eform” option (which causes the coefficient to be displayed in exponentiated form), we obtained the output on the ratio scale:

gen lnOR = ln(OR) gen lnlci = ln(lci) gen lnuci = ln(uci)

The log-transformed values were then used to perform the meta-analyses using the following command:

metan lnOR lnlci lnuci, random efform effect(OR)

# Table S1. Studies’ inclusion and exclusion criteria

The data shown in Table S1 was extracted- and is presented- as it was reported in the included studies.

| **Author/year** | **Objective** | **Inclusion criteria** | **Exclusion criteria** |
| --- | --- | --- | --- |
| Crum- Cianflone 2009 | We performed a cross-sectional study among a well-characterized ethnically diverse cohort of HIV- infected patients. Our aims were  (1) to determine the prevalence of NAFLD and (2) to identify factors associated with NAFLD among HIV-infected patients  without HCV. | All study participants had confirmed HIV infection by enzyme-linked immunosorbent  assay and Western blot testing. | Patients were excluded from participating in this study if they were younger than 18 years or were pregnant as determined by a positive urine betahuman chorionic gonadotropin test. Patients found to have chronic hepatitis B (n=14), chronic hepatitis C (n=3), or chronic hepatitis B and C (n = 4) were excluded from the analysis. Twenty-one patients who self-reported excessive alcohol use, defined as .140 g ethanol per week for men and >70 g ethanol per week for women,7 were excluded; 1 of the 21 was also excluded for having  chronic hepatitis B. |
| Sterling 2013 | We performed a prospective study to define the spectrum of histology in a cohort of HIV- infected patients with abnormal liver enzimes in the absence of  HCV, HBV, DM, and alcohol use | This is a prospective, cross- sectional cohort study in adult HIV-positive subjects with more than 1 abnormal LE, defined as  1.25 to 5 ULN in AST, ALT, or alkaline phosphatase (ALP), over 6 months seen between 2007 and 2011. All patients tested negative for HCV antibody and RNA, HBV surface antigen, and DNA, had normal A1AT, ceruloplasmin, and were negative for ANA, ASMA, and  AMA. | Additional exclusion criteria included history of DM, alcohol use (>30 g/d in men and 20 g/d in women), hepatic decompensation (prothrombin time prolonged>2s, INR>1.5, ascites, hepatic encephalopathy, serum conjugated bilirubin >3.0), thrombocytopenia (platelets< 80,000), concomitant use of vitamin E, thiazolidinediones, metformin, or insulin, use of medications associated with steatosis and/or SH (amiodarone, methotrexate, corticosteroids, estrogen, and tamoxifen), renal failure (serum creatinine>3.0), and advanced HIV disease with life expectancy <1 year. Alcohol consumption was assessed by Alcohol Use Disorders Identification Test16 and subjects with a score >2  were excluded. |
| Nishijima 2014 | The present study was designed to elucidate the prevalence and associated factors, including D drug use, with NAFLD in Asian patients with HIV-1 infection. | The study population was HIV- infected patients, > 17 years, who underwent routine abdominal ultrasonography conducted by certified medical technologists at the Physiological Examination Unit of the hospital, between January 1, 2004 and  March 31, 2013. | The following exclusion criteria were employed in this study; 1) HCV or hepatitis B virus (HBV) infection defined by positive hepatitis C antibody or positive hepatitis B surface antigen, respectively, 2) use of injection drugs, 3) hemophilia, because all HIV-infected hemophiliacs in Japan were exposed to HCV through contaminated blood products, and 4) alcohol consumption of  .20 g of ethanol per day for males and  .10 g/day for females. |
| G Lui 2016 | In this study, we aimed to investigate liver fibrosis and fatty liver in asymptomatic HIV- monoinfected individuals in an  Asian cohort | Inclusion criteria were: (i) positive HIV antibody test, (ii) age  ≥18 years, and (iii) Asian  ethnicity. | Exclusion criteria were: (i) positive hepatitis B surface antigen (HBsAg) or anti-hepatitis B core antibody, (ii) positive HCV antibody, and (iii) refusal to  consent. |
| Kardashian 2017 | We evaluated the contributions of HIV and sex to steatosis, after adjustment for demographic, lifestyle, body composition, and  metabolic factors using data | HIV monoinfected patients from the WIHS cohort | Patients with evidence of hepatitis B surface antigenemia, prior HCV treatment, history of decompensated cirrhosis, metal in their body, or a wide  abdominal girth that would not allow |

|  | collected from two ethnically diverse cohorts of HIV-infected and uninfected adults. |  | them to fit into the MRI scanner were excluded from enrollment. For our analysis, HCV-infected participants from both studies were excluded to examine the relationship of HIV and sex with steatosis in the absence of viral hepatitis  infection. |
| --- | --- | --- | --- |
| Lombardi 2017 | In this study, we retrospectively  evaluated the prevalence and predictors of liver fibrosis in a cohort of HIV-mono-infected patients with persistently elevated transaminases, using simple serum noninvasive fibrosis panels, namely the FIB4 and APRI scores. In addition, we assessed the presence of hepatic steatosis and associated features in this  cohort. | HIV-mono-infected patients who attended the HIV dedicated outpatient service at Royal Free Hospital, London, UK, between January and December 2014. A clinical database was used to identify patients with persistently elevated transaminases in at least two measurements six months apart. | Patients with evidence of HCV or HBV co-infection, as well as other documented causes of liver disease other than NAFLD were excluded. |
| Price 2017 | The aim of this study was to  evaluate associations of adipokines and inflammatory biomarkers with fatty liver in HIV- infected and HIV-uninfected men in the MACS. We hypothesized that (1) adiponectin and inflammatory biomarkers would be associated with fatty liver disease independent of VAT and  (2) the relationship of these markers with fatty liver disease  would differ by HIV serostatus. | Cross-sectional study within the MACS, an ongoing prospective cohort study of men who have  sex with men. | After excluding men who consumed 3 or more alcoholic drinks per day, were infected with hepatitis C or hepatitis B virus, or were missing key covariate data including adipokine or biomarker level testing, 526 men were included in the final fatty liver and biomarker level  analysis |
| Aepfelbacher 2019 | Using transient elastography, a  noninvasive liver ultrasound technology, we characterize hepatic steatosis and fibrosis in a cohort of PWH since early life and assess the relationships between these observations and metabolic and HIV-specific  clinical characteristics. | Forty-six PWH since birth or early childhood were prospectively recruited as part of a convenience sample from a natural history study exploring the physical and psychological impact of lifelong HIV infection. | Participants with known diabetes or active hepatitis C or B virus, and those who were pregnant or were unable to complete transient elastography due to implantable metal devices were excluded from the study. |
| Lallukka- Brück 2019 | We examined 41 HIV+ patients at  baseline and after a 16-year follow-up. The aim was to determine the natural course of NAFLD (LFAT by 1H-MRS) and  diabetes in HIV+ patients with  and without LD. | HIV-monoinfected patients. | NR |
| Vujanović  2019 | The aim of this study was to examine visceral fat thickness (VFT) and its relationship to other anthropometric measurements associated with NAFLD in mono- infected HIV-positive subjects on long-standing cART so as to identify those at risk in order to halt progression to NASH, cirrhosis and hepatocellular  carcinoma. | Inclusion criteria: age = 22–50 years, male gender, confirmed HIV-positive status on polymerase chain reaction (PCR), clinically stable, namely good adherence to cART, an unchanged current drug regimen for ≥ 1 year, at least two consecutive suppressed viral loads. | Exclusion criteria: major psychiatric disorder, active opportunistic infection, history of drug dependence according to the Statistical Manual of Mental Disorders, except for nicotine and alcohol consumption <20 g/day, co- infection with hepatitis B or C viruses. |
| Ajmera 2020 | The current study aims to evaluate the diagnostic accuracy of CAP for the diagnosis of HIV- associated NAFLD and to identify an optimal disease-specific threshold for detection of | Inclusion criteria were as follows:  adult patients at least 18 years of age with a history of HIV infection and at least≥1 of the following risk factors for HIV- associated NAFLD;  hypertriglyceridemia (>150 | Exclusion criteria included serologic  testing to exclude the presence of other forms of liver disease and are outlined in the supplemental material. Further exclusion criteria included alcohol intake of more than 30 g/day in the previous 10  years or greater than 10 g/day in the |

|  | elevated liver fat using MRI-PDFF as the reference standard. | mg/dL), dyslipidemia (low density lipoprotein (LDL) >160 mg/dL or high density lipoprotein (HDL)  <40 mg/dL), serum ALT above the upper limit of normal (>19 U/L for women and >30 U/L for men), body mass index (BMI) >25 kg/m2, hyperuricemia, prediabetes or diabetes defined by American Diabetes  Association criteria. | previous year, evidence of cirrhosis based on clinical assessment or imaging, active illicit drug use, pregnancy, evidence of hepatocellular carcinoma, ingestion of drugs known to cause hepatic steatosis, or inability to undergo MRI. |
| --- | --- | --- | --- |
| Ferri Pezzini 2020 | The aim of this study was to  evaluate the prevalence and risk factors of hepatic steatosis among stable HIV infected patients undergoing ART. Moreover, use of TE was explored as a mean to identify a subgroup of individuals at risk for NASH and/or liver fibrosis to  undergo liver biopsy. | Patients ≥18 years old with undetectable HIV viral load (<50 copies/mL) for at least 12 months were included. | Exclusion criteria were the following: pregnancy; alcohol intake above ≥20 g/day and co-infection with hepatitis B or C viruses. |
| Kaplan 2020 | We therefore aimed to compare  the cardiometabolic profiles and CVD prevalence in PWH with NAFLD to PWH without NAFLD. We hypothesized that compared to PWH alone, PWH with NAFLD would have less favorable cardiometabolic profiles and more prevalent CVD, even after controlling for traditional CVD risk factors and that CVD would  be associated with NAFLD. | We performed a cross-sectional study comparing PWH with NAFLD to PWH without NAFLD. Data were extracted using the Partners Research Patient Data Registry and electronic medical records. In the current study, individuals were identified by an ICD-9 diagnosis of HIV from 2010 to 2017. | Individuals who had not been seen in the Partners Healthcare system for 10 years, with 20% missing data or with less than 2 outpatient visits per year were excluded. Adults with other forms of chronic liver disease, including hepatitis B or C infection and those with significant alcohol use (20 g daily for men or 10 g daily for women) were also excluded. |
| Kirkegaard 2020 | The aim of this study was to  determine whether the prevalence of hepatic steatosis was different between PWH and matched HIV-uninfected individuals. We hypothesized that PWH had a higher prevalence of hepatic steatosis compared with HIV-uninfected individuals. Factors associated with hepatic steatosis were assessed in PWH, and the influence of HIV infection was  evaluated. | PLHIV included in the COCOMO study | Participants were excluded due to age below 40 years (n = 191), CT scan unavailability (n = 143), HBV (n = 23), HCV (n = 82), excessive alcohol consumption (n = 174), or missing information on these parameters (n =  52). |
| Rasoulinejad 2020 | This study was conducted to evaluate liver fibrosis and potential risk factors for PLWH under an ART intervention. | HIV patients undergoing ART  treatment were enrolled to this study and referred for fibro scan after completing the necessary information by completing the  questionnaire and lab tests. | The exclusion criteria were alcohol consumption, hepatitis B and C co infection. |
| Bischoff 2021 | We performed a longitudinal  prospective observational study to screen for development or progression of steatosis as a hallmark of NAFLD and identify related risk factors for the emergence of hepatic steatosis in  PLHIV. | Eligibility criteria comprised an age >18 years, a confirmed HIV diagnosis and a self-reported alcohol intake of less than 30 g/day for male participants and 20 g/day for female participants. | Patients with concomitant viral hepatitis B (HBV) or C (HCV) coinfection were excluded. |
| Fonseca de Almeida 2021 | The aim of this study was to evaluate the relationship between dietary fatty acid intake and NAFLD and/or the presence | All participants with HIV infection enrolled in the PROSPEC-HIV study from June 2015 to January 2019 were eligible for this  analysis. | Participants with viral hepatitis co- infection defined by positive HCV- antibody or positive HBsAg; excessive alcohol consumption defined by the  Alcohol Use Disorders Identification Test |

|  | of liver fibrosis in HIV mono- infected individuals. |  | (AUDIT) score _8; use of lipids supplements or missing laboratory/inconsistent data on dietary  assessment were excluded. |
| --- | --- | --- | --- |
| Jongraksak 2021 | The primary objective of this study was to evaluate the prevalence of MAFLD in PLWH. The secondary objectives were to determine the predicting factors of MAFLD and severity of MAFLD  in PLWH | The main inclusion criteria were PLWH who were receiving ART with an undetectable HIV viral load for at least 6 months and signed consent to participate in  the study. | The exclusion criteria included patients  with the presence of hepatitis B virus or hepatitis C virus co-infection, other known liver diseases such as cirrhosis or hepatocellular carcinoma, critical illness or active opportunistic infection, and active alcohol consumption (male >30  g/day, female>20 g/day). |
| Liu Danping 2021 | In this study, we aimed to explore the prevalence of MAFLD and to determine the risk factors related to MAFLD in PLWH in absence of HBV or HCV infection and significant alcohol intake. | The inclusion criteria for this cohort included HIV+ persons, the age of 18~70 years and who had written informed consent obtained. | Exclusion criteria included the following:  coinfection with HBV or HCV; secondary causes of fatty liver (e.g., consumption of amiodarone and tamoxifen) and decompensated liver disease; significant alcohol consumption (>20 g per day for man, >10g per day for woman); receipt of antibiotics in the preceding two months; autoimmune disease; severe heart, lung, kidney, brain, blood diseases or any other significant systemic diseases and pregnant or lactating  women. |
| Arka De 2022 | We aimed to study the prevalence, severity and risk factors of NAFLD in a well- characterized cohort of Indian  patients with HIV infection. | Consecutive adult patients’ mono-infected with HIV and attending the ART centre of an academic medical centre in north India over a duration of 1-year  were recruited | Patients with significant alcohol  consumption (>30 g/day in males and  >20 g/day in females), co-infection with Hepatitis B virus or Hepatitis C virus, other co-existent etiologies of liver disease, use of steatogenic drugs apart from ART, hepatocellular carcinoma or other malignancies, and WHO stage IV  HIV infection were excluded |
| Busca 2022 | The objectives of our study were to compare various tests for diagnosis of steatosis and fibrosis with liver biopsy in PWH with  increased transaminase levels. | The study population included all  PWH seen at Hospital Universitario La Paz, Madrid, Spain with increased transaminase levels for ≥6 months (confirmed by ≥2 samples) from January 2017 to June 2018. Other inclusion criteria were being on stable combination ART and having HIV- ribonucleic acid<50 copies/mL for  ≥1 year | The exclusion criteria were past or present chronic HBV or active HCV coinfection, high alcohol consumption (>30 g/day in men or ≥20 g/day in women), potential drug hepatotoxicity,  and other liver diseases. |
| Lemoine 2022 | We aimed to assess (1) the prevalence, severity, and risk factors of liver steatosis and AF in moninfected PLWH at risk of NAFLD; and (2) the performance of the CAP technique and its best cutoff for the diagnosis of moderate to severe steatosis in  this population. | Between March 2014 and  November 2015, HIV-1 infected individuals over 40 years, receiving antiretroviral treatment (ART) for at least 5 years with HIV viral load <400 copies/mL and CD4-T cell count >100/mm3, were invited to participate to the study if they met at least 1 of the following criteria: (1) MetS defined by the 2009 international criteria19 (Group 1); (2) persistently elevated liver enzymes defined by transaminases≥1.5 upper limit of normal ([ULN] ¼ 35 IU/mL) and/or gammaglutamyltransferase level 2 ULN (ULN ¼ 60 IU/L) on 2 blood  samples within at least a 3- | Participants were not eligible if they met  one of the following criteria: positive hepatitis B or C virus serologies; coinfection with HIV-2; use of intravenous drugs within the last 6 months; current or past excessive alcohol intake (>30 g/day); genetic hemochromatosis; autoimmune hepatitis; primary or secondary biliary cirrhosis or cholangitis; alpha1 antitrypsin deficiency; Wilson’s disease; secondary causes of NAFLD (ie, ongoing prolonged steroid therapy, current therapy with amiodarone, tamoxifen, methotrexate, nifedipine, or hycanthone, history of cancer chemotherapy; short bowel syndrome; polycystic ovarian syndrome; Weber- Christian disease); active opportunistic  infection except for candida |

|  |  | month interval (Group 2); or (3) clinical lipodystrophy as  previously described | oesophagitis; ongoing cancer; pregnancy; or uncontrolled congestive  heart failure. |
| --- | --- | --- | --- |
| Michel 2022 | The aim of this prospective study  was to determine differences in HRQL between PLWH with and without HS, and to identify treatable predictors of an  impaired HRQL in both groups. | People living with HIV. | NR |
| Sebastiani 2022 | This study aims to investigate if  five simple serum biomarkers applied to PWH can optimize a two-tier care pathway for identifying significant liver fibrosis as defined by TE. Specifically, we evaluate the reduction in the TE referral rate and related costs that would have occurred based on biomarker assessment, and the discordance rate between TE and simple fibrosis biomarkers.  Finally, we determine the factors associated with the discordance between serum biomarkers and  TE. | We included all consecutive patients with HIV infection (documented by positive enzyme-linked immunosorbent assay (ELISA) with Western blot  confirmation) age ≥ 18 years with availability of TE with CAP and relevant clinical and biochemical parameters. | Exclusion criteria were: (i) positivity for HCV antibody or hepatitis B surface antigen; (ii) evidence of other liver disease; (iii) significant alcohol intake, defined as more than 30g/day in men and more than 20g/day in women; (iv) history of HCC or liver transplantation;  (v) contraindications (pregnancy, pacemaker insertion) and failure or unreliable measurement of TE examination with CAP; and (vi) missing liver transaminases or platelets. |

# Table S2. Characteristics and reasons for exclusion of key studies that were not included in the systematic review.

| **Study ID** | **Included in the SR by Maurice et**  **al.?** | **Title** | **Source** | **Inclusion?** | **Comments** |
| --- | --- | --- | --- | --- | --- |
| Lemoine 2006 | Yes | Altered hepatic expression of SREBP-1 and PPARgamma is associated with liver injury in insulin- resistant lipodystrophic HIV-  infected patients | AIDS. 2006  Feb 14;20(3):387-  95. doi: 10.1097/01.ai ds.000020650 3.01536.11. | Excluded | Risk of overlapping primary study data with **Lemoine 2022** |
| Guaraldi 2008 | Yes | Nonalcoholic fatty liver disease in HIV- infected patients referred to a metabolic clinic: prevalence, characteristics, and  predictors | Clin Infect Dis. 2008 Jul  15;47(2):250-  7. doi: 10.1086/5892 94. | Excluded | Risk of overlapping primary study data with **Sebastiani 2022** |
| Ingiliz 2009 | Yes | Liver damage underlying unexplained transaminase  elevation in human | Hepatology. 2009 Feb;49(2):436  -42. doi: | Excluded | Risk of overlapping primary study data with **Lemoine 2022** |

|  |  | immunodeficiency virus-1 mono-infected patients on  antiretroviral therapy | 10.1002/hep.  22665. |  |  |
| --- | --- | --- | --- | --- | --- |
| Vodkin 2015 | Yes | Clinical, biochemical and histological differences between HIV-associated NAFLD and primary NAFLD: a case-control study | Aliment Pharmacol Ther. 2015 Feb;41(4):368  -78. doi: 10.1111/apt.  13052. | Excluded | In this study, all PLHIV had NAFLD and were compared to NAFLD patients without HIV. Therefore, the primary outcome could not be estimated with the data provided. Moreover, this study was at risk of overlapping primary study  data with **Ajmera 2021** |
| Morse 2015 | Yes | Nonalcoholic Steatohepatitis and Hepatic Fibrosis in HIV-1-Monoinfected Adults With Elevated Aminotransferase Levels on  Antiretroviral Therapy | Clin Infect Dis. 2015  May 15;60(10):156  9-78. doi: 10.1093/cid/c iv101. | Excluded | Based on the population they included and the methodology they reported, it was unclear if they excluded individuals with a history of viral hepatitis. In addition, the study was at risk of overlapping primary study data with **Aepfelbacher 2019** |
| Vuille- Lessard 2016 | Yes | Nonalcoholic fatty liver disease diagnosed by transient elastography with controlled attenuation parameter in unselected HIV monoinfected patients | AIDS. 2016  Nov 13;30(17):263  5-2643 | Excluded | Risk of overlapping primary study data with **Sebastiani 2022** |
| Lemoine 2019 | No | Diagnostic Accuracy of Noninvasive Markers of Steatosis, NASH, and Liver Fibrosis in HIV-Monoinfected Individuals at Risk of Nonalcoholic Fatty Liver Disease (NAFLD): Results From the  ECHAM Study | J Acquir Immune Defic Syndr. 2019 Apr 1;80(4):e86- e94. | Excluded | Risk of overlapping primary study data with **Lemoine 2022** |
| Maurice 2021 | No | Increased Body Mass Index and Type 2 Diabetes Are the Main Predictors of Nonalcoholic Fatty Liver Disease and Advanced Fibrosis in Liver Biopsies of Patients With Human Immunodeficiency  Virus Monoinfection | Clin Infect Dis. 2021 Oct 5;73(7):e2184  -e2193 | Excluded | Risk of overlapping primary study data with **Ajmera 2021, Lombardi 2017, and**  **Sebastiani 2022** |
| Price 2022 | No | Human Immunodeficiency Virus Is Associated With Elevated FibroScan–Aspartate Aminotransferase  (FAST) Score | Clin Infect Dis. 2022 Dec  19;75(12):211  9-2127. doi:  10.1093/cid/c iac337. | Excluded | Included patients with a history of hepatitis C virus infection. In addition, the study did not report data on the outcomes of interest using the predefined definitions of our systematic review. |

1. **Figure S4. Subgroup analysis by type of study design (for the meta- analysis of prevalence)**


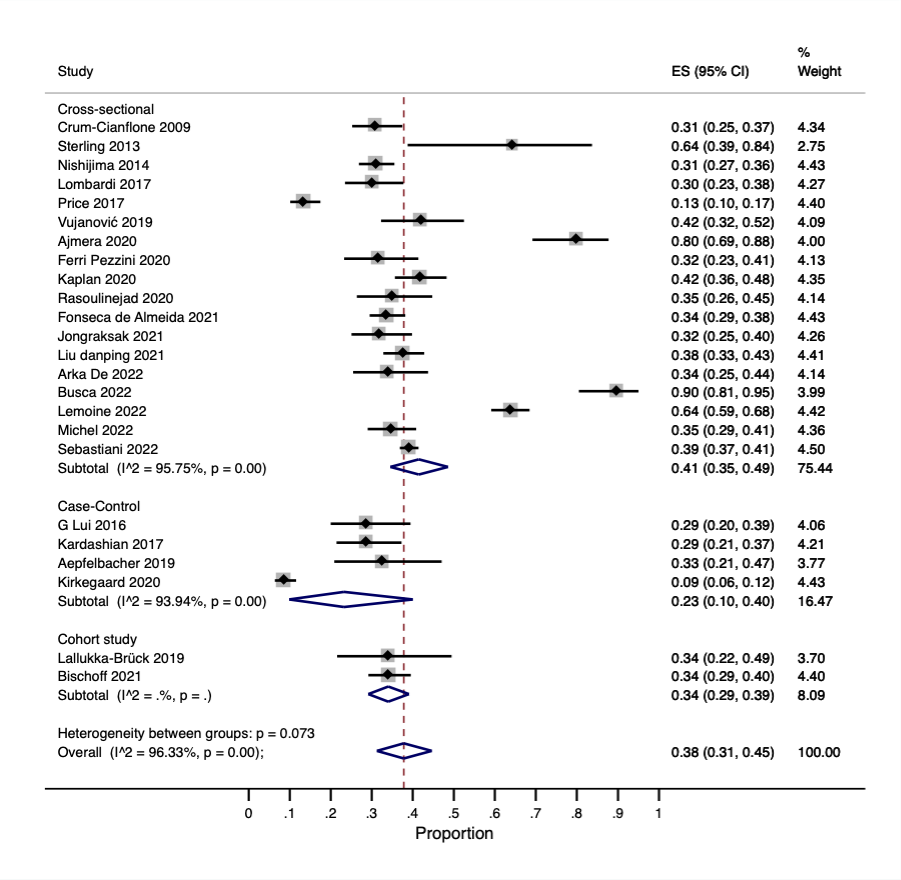


# Figure S5. Meta-analysis of adjusted ORs of the association between Age and NAFLD in PLHIV


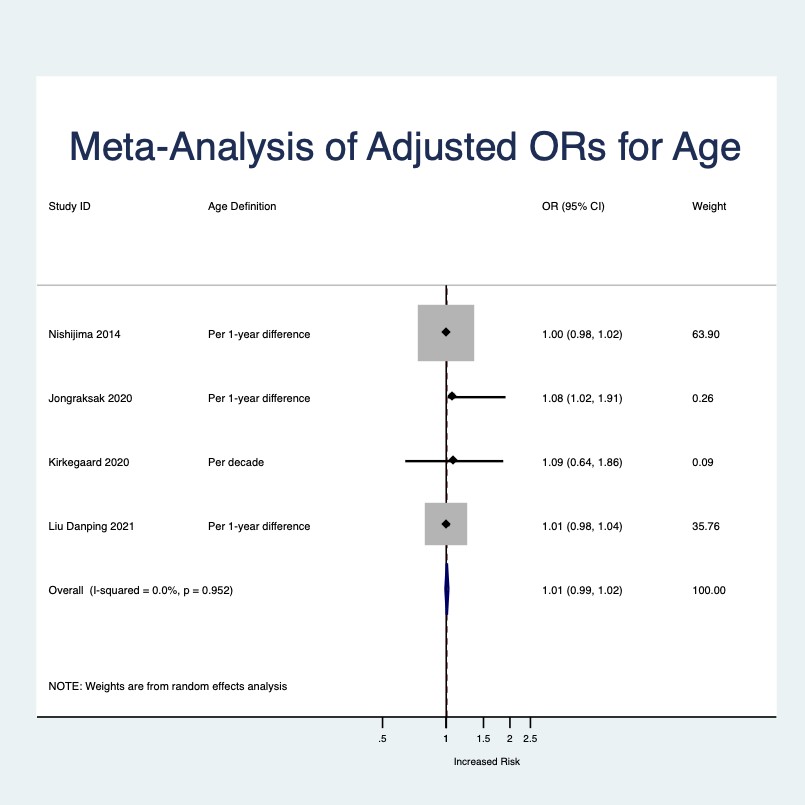

Supplement: Supplementary file 1 — Supporting Information [file JIA2-26-e26072-s001.docx]
